# In Silico Design of Natural Inhibitors of ApoE4 from the Plant *Moringa oleifera*: Molecular Docking and Ab Initio Fragment Molecular Orbital Calculations

**DOI:** 10.3390/molecules28248035

**Published:** 2023-12-11

**Authors:** Divya Shaji, Yoshinobu Nagura, Haruna Sabishiro, Ryo Suzuki, Noriyuki Kurita

**Affiliations:** Department of Computer Science and Engineering, Toyohashi University of Technology, Tempaku-cho, Toyohashi 441-8580, Aichi, Japan

**Keywords:** Alzheimer’s disease, ApoE4, natural inhibitors, *Moringa oleifera*, quercetin, fragment molecular orbital, molecular docking, molecular mechanics, in silico drug design

## Abstract

Alzheimer’s disease (AD) is a neurological disease, and its signs and symptoms appear slowly over time. Although current Alzheimer’s disease treatments can alleviate symptoms, they cannot prevent the disease from progressing. To accurately diagnose and treat Alzheimer’s disease, it is therefore necessary to establish effective methods for diagnosis. Apolipoprotein E4 (ApoE4), the most frequent genetic risk factor for AD, is expressed in more than half of patients with AD, making it an attractive target for AD therapy. We used molecular docking simulations, classical molecular mechanics optimizations, and ab initio fragment molecular orbital (FMO) calculations to investigate the specific interactions between ApoE4 and the naturally occurring compounds found in the plant Moringa Oleifera. According to the FMO calculations, quercetin had the highest binding affinity to ApoE4 among the sixteen compounds because its hydroxyl groups generated strong hydrogen bonds with the ApoE4 residues Trp11, Asp12, Arg15, and Asp130. As a result, we proposed various quercetin derivatives by introducing a hydroxyl group into quercetin and studied their ApoE4 binding properties. The FMO data clearly showed that adding a hydroxyl group to quercetin improved its binding capacity to ApoE4. Furthermore, ApoE4 Trp11, Asp12, Arg15, and Asp130 residues were discovered to be required for significant interactions between ApoE4 and quercetin derivatives. They had a higher ApoE4 binding affinity than our previously proposed epicatechin derivatives. Accordingly, the current results evaluated using the ab initio FMO method will be useful for designing potent ApoE4 inhibitors that can be used as a candidate agent for AD treatment.

## 1. Introduction

Alzheimer’s disease (AD) is a degenerative condition that causes the deterioration of brain cells, resulting in dementia characterized by declining cognitive function and decreased ability to perform daily activities. Several risk factors have been identified, including advanced age, genetic predisposition, head injuries, vascular disorders, infections, and environmental factors [1]. Currently, the number of AD patients globally is reported to be around 50 million, and this number is projected to increase rapidly in the coming years, raising concerns about the impact on families and the economy [1]. Currently, the pharmacological care of AD is confined to the application of cholinesterase inhibitors and N-methyl-d-aspartate antagonists, which are used to relieve symptoms rather than cure or prevent the disease [1]. Recent studies have focused on using multi-omics analyses to identify natural compounds and metabolites with neuroprotective properties that can modulate signaling pathways related to neurovascular endothelial cells in AD treatment [2].

Less than 5% of Alzheimer’s cases are early-onset AD, whereas late-onset AD (LOAD) refers to a large portion of Alzheimer’s cases that occur after the age of 65 [3]. The likelihood of LOAD is substantially raised by a mutation in the apolipoprotein E (ApoE) gene, which has three major allele variants (ε2, ε3, and ε4). The major risk component for LOAD is the ApoE4 allele, while ApoE2 appears to confer protection against AD [4,5,6,7,8]. ApoE is an essential cholesterol transporter that contributes to lipid transfer and brain regeneration. Moreover, ApoE4 is linked with a greater possibility of cerebral amyloid angiopathy and age-associated dementia [8].

Natural products have historically played a crucial role in the discovery of pharmaceuticals, particularly for the treatment of infectious and malignant diseases, as well as other medical conditions such as cardiovascular disease and multiple sclerosis [9]. Here, we examined the interactions between ApoE4 and various substances found in the plant *Moringa oleifera* (*M. oleifera*) to identify potent natural inhibitors of ApoE4. *M. oleifera* is a member of the *Moringaceae* family, which originated in the Indian subcontinent [10] and is commonly referred to as the “miracle tree” or “tree of life” due to its widespread use as a functional food and nutritional supplement [11]. For example, its plant parts such as leaves, roots, bark, gum, flowers, fruits, seeds, and seed oil contain biologically active substances such as vitamins, carotenoids, polyphenols, phenolic acids, flavonoids, alkaloids, glucosinolates, isothiocyanates, tannins, and saponins, which are believed to have high nutritional and therapeutic effects [10,12,13]. Accordingly, numerous pharmacological properties of *M. oleifera*, including antibacterial, anti-hypercholesterolemic, antitumor, antidiabetic, anti-inflammatory, and antioxidant properties, have been reported [10,11,14,15,16,17,18,19]. Recent research has shown that *M. oleifera* has neuroprotective properties against the symptoms of AD, dementia, Parkinson’s disease, stroke, and neurotoxicity [11,16,20,21,22,23,24,25,26,27,28].

In a recent study [29,30], we investigated the binding characteristics of the SARS-CoV-2 main protease (Mpro) and 35 compounds that occur naturally from *M. oleifera*, *Aloe vera*, and *Nyctanthes arbour-tristis* using a combination of molecular simulation approaches. To discover which compound binds deeply to Mpro and to propose new medicinal compounds that could serve as possible Mpro inhibitors, we performed protein–ligand docking simulations, classical molecular mechanics (MM) optimizations, and ab initio fragment molecular orbital (FMO) computations. Using the same molecular simulations, we recently studied the interactions between ApoE4 and compounds from the spice cinnamon [31]. Epicatechin was found to have the greatest potential to bind to ApoE4 of the ten natural compounds. In addition, we proposed various epicatechin derivatives and investigated their interactions with ApoE4. We found that adding a hydroxyl group at an appropriate position of epicatechin greatly improved its ability to bind ApoE4. Furthermore, it was shown that ApoE4’s Asp130 and Asp12 residues play a vital role in the binding of ApoE4 to epicatechin derivatives [31].

Using similar ab initio molecular simulations as in our previous research [29,30,31], here, we investigated the ability of natural products contained in *M. oleifera* to bind specifically to ApoE4. The binding properties of 16 natural products from *M. oleifera* with ApoE4 were investigated in detail using the ab initio FMO approach [32]. Quercetin was found to interact most effectively with ApoE4 and thus could be a therapeutic lead molecule as a potent ApoE4 inhibitor. Moreover, we proposed some new quercetin derivatives and investigated their ability to bind to ApoE4. The results of FMO calculations showed that the proposed compounds exhibited higher binding affinity for ApoE4 than the original quercetin as well as our previously reported epicatechin derivatives [31]. Accordingly, it can be assumed that our proposed quercetin derivatives have a considerable inhibitory effect on ApoE4.

## 2. Results and Discussion

### 2.1. Optimized Structures of the ApoE4–Ligand Complexes

**Figure 1:**
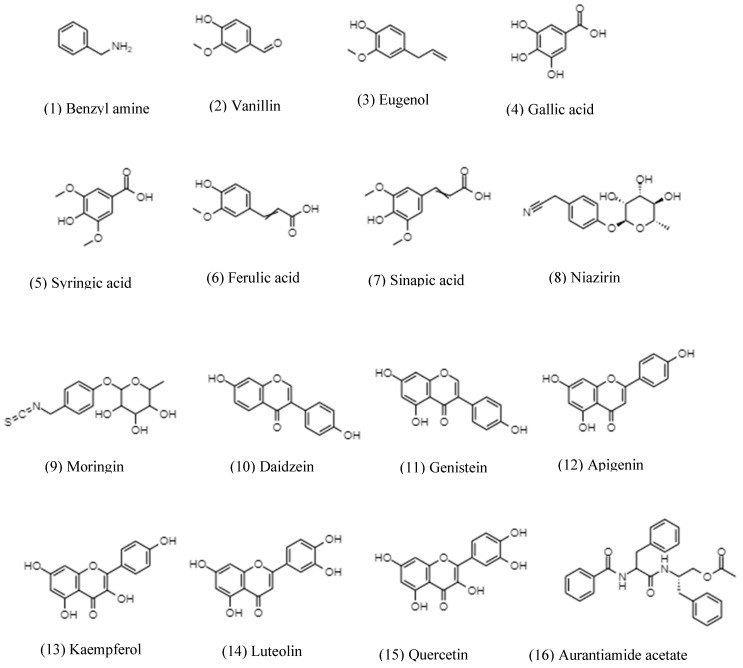
Chemical structures of our used compounds contained in *Moringa oleifera*.

The chemical structures of the compounds used in the present study are depicted in Figure 1. Table S1 of the Supporting Information lists their pharmacokinetic characteristics computed with the SwissADME web tool [33]. According to Lipinski’s rule of five [34], active drugs must meet the following criteria: (1) the molecular weight (MW) is less than 500 g/mol, (2) the number of H-bond acceptors is less than 10, (3) the number of H-bond donors is less than five, and (4) the octanol–water partition coefficient (LogP) is less than 5. As indicated in Table S1, 16 compounds satisfied Lipinski’s rule [34]. Other significant properties such as total polar surface area (TPSA) and the number of rotatable bonds were also estimated using SwissADME [33]. TPSA and the number of rotatable bonds for orally active drugs should be less than 140 Å^2^ and 10, respectively. As listed in Table S1, all compounds satisfy the conditions. Therefore, we used these compounds and optimized their structures using Gaussian 16 [35] to determine their restrained electrostatic potential (RESP) charges [36].

Using the protein–ligand docking simulation program AutoDock 4.2.6 [37], we docked each compound to the ligand-binding pocket of ApoE4 based on the optimized structure and the RESP charge of the compound. Figure S1a shows the surface representation of the optimized ApoE4 structure and its ligand-binding cavity, demonstrating that ApoE4 contains a large binding cavity for ligand docking. The generated candidate structures of the ApoE4–compound complex were grouped into clusters according to their structural similarity. These clusters were ranked based on the size of the lowest binding energy (BE) between ApoE4 and the compound for the structures contained in each cluster. Since the classical MM approach was used to assess BEs, some inaccuracies may exist in these values. Therefore, the AutoDock results were only used to choose the representative structures of the ApoE4–compound complexes for further computations. Table 1 lists the cluster ranking, BE, and number of poses for the cluster with the largest number of poses. Among the clusters generated with AutoDock 4.2.6 [37], we here used the cluster with the largest number of poses because there is a high probability for a compound to have one of the structures in the cluster with the largest number of poses.

To optimize the candidate structures of the ApoE4–compound complex, we used the MM method [38,39,40] of the AMBER18 (Assisted Model Building with Energy Refinement) program [41]. The structures were fully optimized in explicit water molecules, and quercetin was confirmed to bind to the ligand-binding pocket of ApoE4, as shown in Figure S1b. For the other compounds, similar structures bound to the ligand-binding pocket were obtained with the MM optimizations. To validate the accuracy of the docking simulations, we conducted a re-docking simulation and analyzed the root mean square deviation (RMSD) between the docked and the crystallized conformations of the native ligand in the ligand-binding site of ApoE4. Figure S2 shows the superimposed structures of the docked and the crystallized conformations. The obtained RMSD between both conformations is 0.29 Å, indicating the accuracy of our docking simulations. Therefore, we used the same docking and MM-optimization methods for obtaining the stable structures of the ApoE4 complexes with other compounds.

### 2.2. Interactions between ApoE4 and Compounds with Higher Binding Affinities

To determine which compound binds most strongly to ApoE4, we first investigated the total inter-fragment interaction energy (IFIE) between the compound and all ApoE4 residues for the MM optimized structure using the ab initio FMO method [32]. Table 2 lists the total inter-fragment interaction energies (IFIEs) for the 16 compounds along with the ApoE4 residues participating in the hydrogen bond interactions with each compound. A larger magnitude of the total IFIE indicates a stronger interaction between ApoE4 and the compound. Of the 16 compounds, quercetin has the largest total IFIE (−136.2 kcal/mol). Its magnitude is 26.7 kcal/mol larger than that of the second-best compound luteolin. Furthermore, the total IFIE of quercetin is 16.5 kcal/mol higher than that of epicatechin, which was determined to be the top-ranked compound as an ApoE4 inhibitor in our previous study [31]. Accordingly, it is expected that quercetin will be a potent inhibitor of ApoE4.

The flavonoid quercetin is contained in the leaves of *M. oleifera* [42] at a concentration of 384.61 mg/100 g [43]. Quercetin is responsible for Moringa’s hypolipidemic, antihypertensive, antioxidant, and anti-diabetic activities [44]. It protects the body against neurotoxic substances and can prevent the development of neuronal damage and dementia [45]. Many in vitro and in vivo studies have proven that quercetin has strong anti-inflammatory properties [46]. According to a previous study, quercetin showed a protective effect against synucleinopathies by effectively preventing alpha-synuclein aggregation [47]. In the brains of AD patients, tau protein is abnormally misfolded at both its presynaptic and postsynaptic terminals. This abnormal tau is abundant in synaptoneurosomal fractions [48]. It has been reported that quercetin can significantly reduce tau protein hyper-phosphorylation [49]. Acetylcholin esterase (AChE) inhibitors enhance nicotinic receptor expression in AD patients, which improves cognitive memory [50]. Previous studies have demonstrated that quercetin is very effective at inhibiting AChE [51,52,53]. In AD mice, quercetin has been shown to inhibit the accumulation of Aβ by balancing ApoE4 [54]. Therefore, quercetin is expected to significantly contribute to the treatment of AD by stabilizing ApoE4 [55].

**Figure 2:**
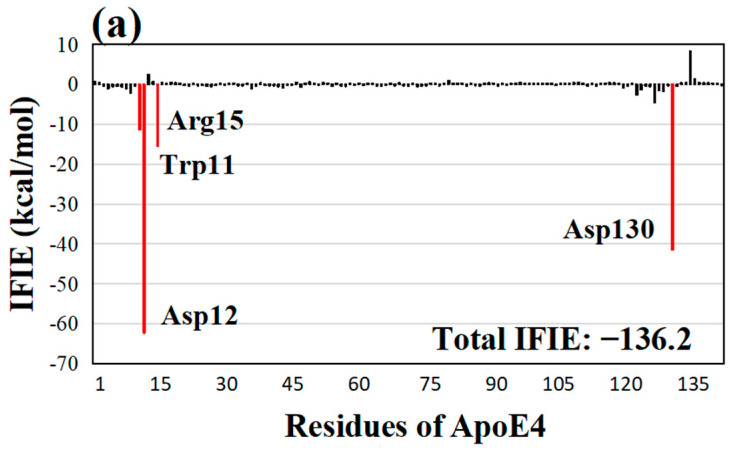

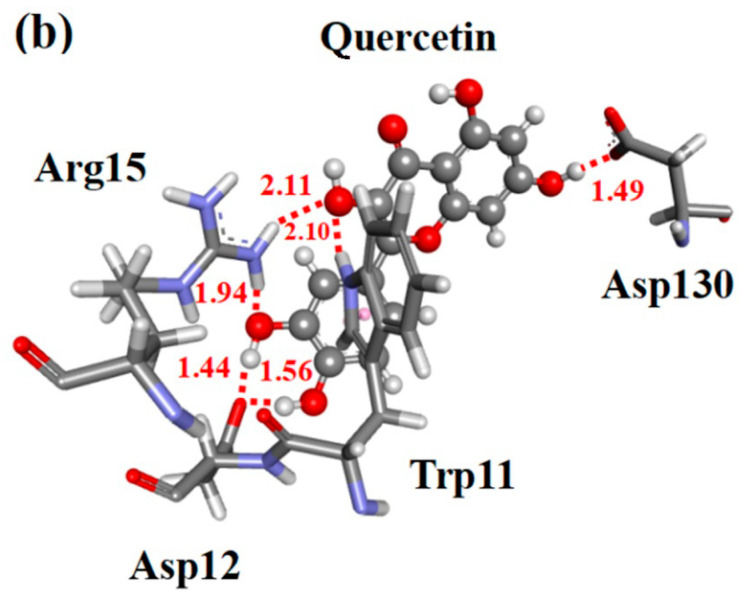
(**a**) IFIEs between the best compound quercetin and ApoE4 residues. The total IFIE of quercetin is also shown. ApoE4 residues with negative IFIEs greater than 10 kcal/mol are displayed with red bars. (**b**) Structure of the interactions between quercetin and key ApoE4 residues. Hydrogen bonds are represented by red dotted lines, and their distances are indicated in Å.

To elucidate why quercetin binds most strongly to ApoE4, we examined the IFIEs between each ApoE4 residue and compound evaluated using the ab initio FMO method. Figure 2a shows that quercetin strongly interacts with the Asp12 and Asp130 residues of ApoE4, indicating that these two residues are responsible for the strong binding between ApoE4 and quercetin. The magnitudes of IFIEs for the residues Trp11 (−11.4 kcal/mol), Asp12 (−62.3 kcal/mol), Arg15 (−15.4 kcal/mol), and Asp130 (−41.4 kcal/mol) are larger than 10 kcal/mol. The strong binding of quercetin is considerably facilitated by Asp12, as it has a very high IFIE value of −62.3 kcal/mol. We also investigated the interaction structure between these ApoE4 residues and quercetin. As seen in Figure 2b, quercetin formed six hydrogen bonds with the ApoE4 residues. Two strong hydrogen bonds were created between the oxygen atom of Asp12 and the hydrogen atoms of the two distinct hydroxyl groups of quercetin at 1.44 and 1.56 Å, respectively. The other hydroxyl group in quercetin formed a strong hydrogen bond with the oxygen atom in Asp130 at 1.49 Å. The oxygen atoms of the two different hydroxyl groups of quercetin established hydrogen bonds with the hydrogen atoms of the NH_2_ group of Arg15 at 1.94 and 2.11 Å, respectively. In addition, the hydrogen atom in the NH group of Trp11 and the oxygen atom in the hydroxyl group of quercetin formed a hydrogen bond at 2.10 Å. Notably, four distinct hydroxyl groups in quercetin formed hydrogen bonds with the ApoE4 residues, resulting in a higher total IFIE between quercetin and ApoE4. Furthermore, quercetin formed strong hydrogen bonds with the negatively charged ApoE4 residues Asp130 and Asp12. These charged residues might be hydrated by surrounding water molecules, facilitating the screening of their electrostatic interactions with quercetin. We thus checked the positions of the water molecules, which are hydrogen-bonded to Asp130 or Asp12 of ApoE4 in the MM-optimized structure of the ApoE4−quercetin complex. It was confirmed that Asp12 and Asp130 in the ApoE4−quercetin complex did not have any hydrogen bonds with water molecules. Therefore, the electrostatic interactions between these negatively charged residues of ApoE4 and the positively charged groups of quercetin were not screened by water molecules. As a result, the electrostatic interactions remained essential for the strong binding between ApoE4 and quercetin. Similar interacting properties were obtained for the ApoE4 complexes with the other compounds.

Luteolin had the second-highest total IFIE (−109.5 kcal/mol) among the compounds utilized in the present study, as listed in Table 2. Luteolin is a flavonoid found in Moringa leaves [56]. It is a naturally occurring antioxidant that can be found in various vegetables and spices [57]. Luteolin has been reported to have anti-inflammatory and anti-allergic properties. It can offer protection against conditions like cardiovascular disease that are related to inflammatory processes [57]. Many in vitro and in vivo studies [58,59] have shown that luteolin has excellent neuroprotective characteristics through various pathways. Previous studies [60,61] found that luteolin inhibited aging-related brain microglia activity and neuroinflammation, resulting in improved cognitive functions. Recently, an interesting antiviral activity against the severe acute respiratory syndrome coronavirus 2 (SARS-CoV-2) has been reported [62].

**Figure 3:**
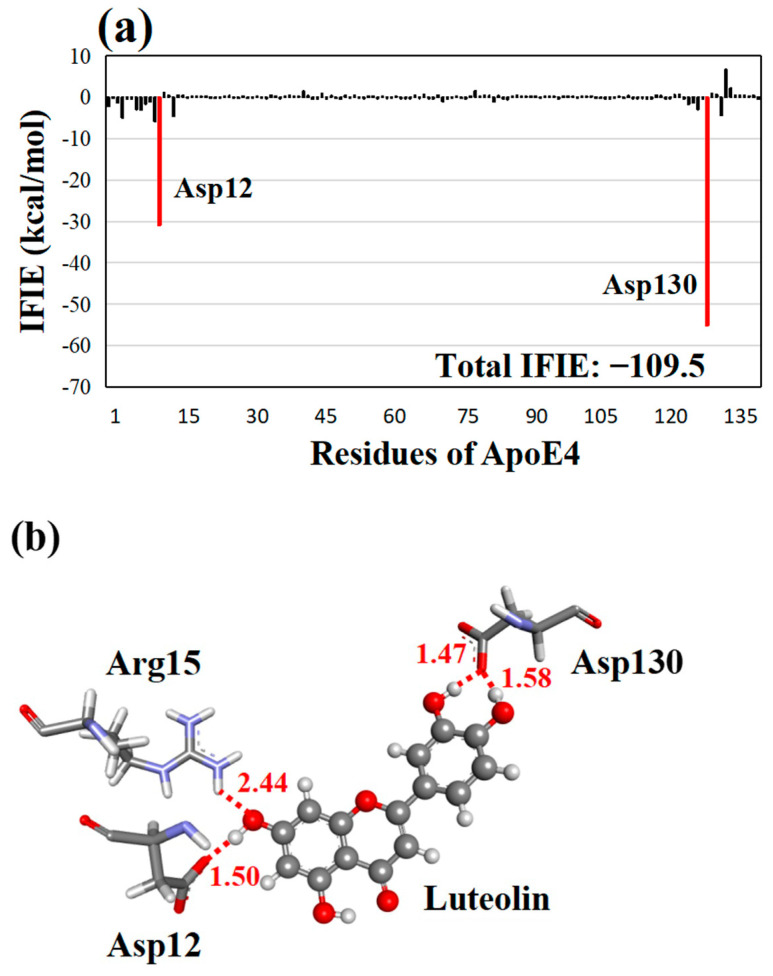
(**a**) IFIEs between the second-best compound luteolin and ApoE4 residues. The total IFIE of luteolin is also shown. ApoE4 residues with negative IFIEs greater than 10 kcal/mol are displayed with red bars. (**b**) Structure of the interactions between luteolin and key ApoE4 residues. Hydrogen bonds are represented by red dotted lines, and their distances are given in Å.

In the present FMO calculations, the strongest favorable interaction (−55.2 kcal/mol) between luteolin and Asp130 of ApoE4 was observed, followed by that between luteolin and Asp12 (−30.9 kcal/mol), as shown in Figure 3a. Therefore, it was revealed that these Asp residues were essential for the strong interaction between ApoE4 and luteolin. These residues formed three hydrogen bonds with luteolin, as seen in Figure 3b. An oxygen atom of Asp130 formed two strong hydrogen bonds with the hydrogen atoms of the two different hydroxyl groups of luteolin at 1.47 and 1.58 Å, respectively. Similarly, the oxygen atom of Asp12 formed a hydrogen bond with the hydrogen atom of a hydroxyl group in luteolin at 1.50 Å. Moreover, the oxygen atom of the same hydroxyl group formed another weak hydrogen bond with the hydrogen atom of Arg15 at 2.44 Å.

As shown in Table 2, genistein had the third-highest total IFIE of the compounds studied, with a value of −109.1 kcal/mol, which was only 0.4 kcal/mol lower than the second-highest compound luteolin. Genistein is an isoflavone found in Moringa leaves, legumes, peanuts, and green peas [63]. It was indicated that genistein has potential as a lead molecule in the treatment of a wide range of diseases, such as postmenopausal symptoms, cancer, and bone, brain, and heart problems. As genistein is considered to pass the blood–brain barrier to exert its neuroprotective effect, it has been extensively studied in the treatment of neurodegenerative diseases, including Alzheimer’s, Huntington’s, and Sanfilippo diseases [63].

**Figure 4:**
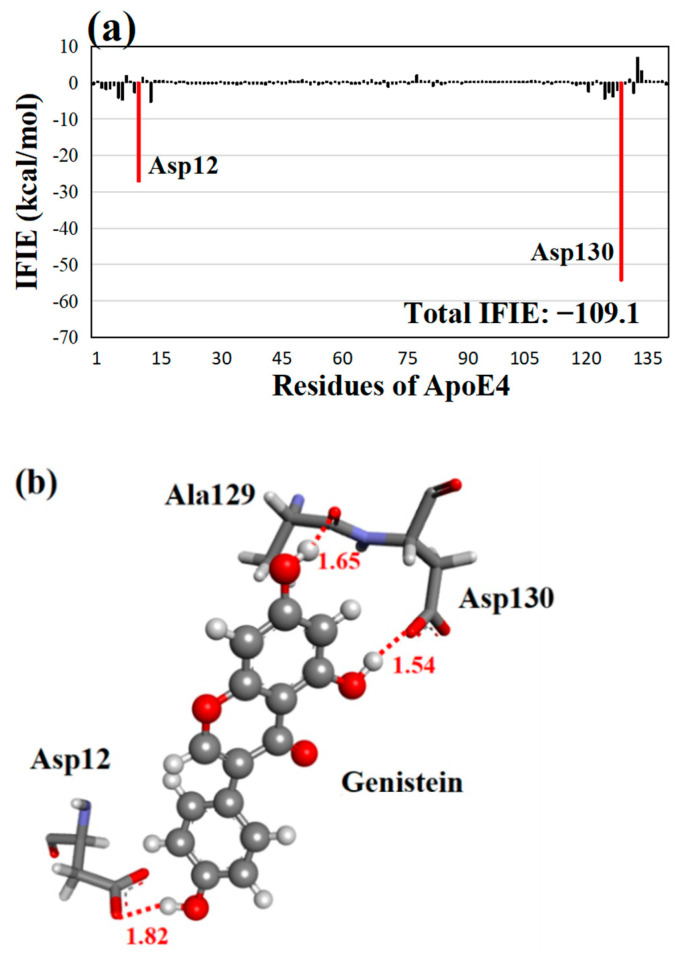
(**a**) IFIEs between the third-best compound genistein and ApoE4 residues. The total IFIE of genistein is also shown. ApoE4 residues with negative IFIEs greater than 10 kcal/mol are displayed with red bars. (**b**) Structure of the interactions between genistein and key ApoE4 residues. Hydrogen bonds are represented by red dotted lines, and their distances are given in Å.

As depicted in Figure 4a, the strongest attractive interaction (−54.3 kcal/mol) was found between genistein and Asp130, followed by genistein and Asp12 (−27.2 kcal/mol), suggesting the importance of these Asp residues for the strong interactions between ApoE4 and genistein. As shown in Figure 4b, these residues formed two hydrogen bonds with genistein. At 1.54 Å, an oxygen atom of Asp130 formed a strong hydrogen bond with a hydrogen atom of the hydroxyl group of genistein. At 1.82 Å, an oxygen atom of Asp12 formed a hydrogen bond with the hydrogen atom of the hydroxyl group of genistein. Furthermore, the oxygen atom of the carbonyl group of the main chain between Ala129 and Asp130 formed a hydrogen bond with the hydrogen atom of a hydroxyl group of genistein at 1.65 Å. Accordingly, as indicated in Figure 2, Figure 3 and Figure 4, Asp12 and Asp130 of ApoE4 were elucidated to be the key residues for the strong binding between ApoE4 and the three compounds (quercetin, luteolin, and genistein) with higher total IFIEs to ApoE4. These residues were also essential for the strong interactions with ApoE4 and cinnamon compounds in our previous study [31].

### 2.3. Interactions between ApoE4 and Other Compounds with Lower Binding Affinities

As indicated in Table 2, only five compounds of the 16 used in our present study had total IFIEs greater than 100 kcal/mol. The total IFIEs for moringin and niazirin were −102.7 and −101.5 kcal/mol, respectively. The distributions of IFIEs and interaction structures between ApoE4 residues and moringin and niazirin are shown in Figures S3 and S4 of the Supporting Information, respectively. As shown in Figure S3a, Asp12 had the strongest interaction with moringin since the oxygen atom of Asp12 formed two strong hydrogen bonds at 1.55 and 1.58 Å with the hydrogen atoms of the two different hydroxyl groups of moringin, as shown in Figure S3b. Additionally, a hydrogen bond was formed between the oxygen atom of the hydroxyl group of moringin and the hydrogen atom of the NH_2_ group of Arg15.

Niazirin had strong attractive interactions with Asp12 (−47.2 kcal/mol) and Arg15 (−26.9 kcal/mol), as shown in Figure S4a. These residues formed five hydrogen bonds with niazirin. The oxygen atom of Asp12 formed two hydrogen bonds with the two distinct hydroxyl groups of niazirin at 1.52 and 1.78 Å, as shown in Figure S4b. In addition, the hydrogen atoms of the NH_2_ group of Arg15 formed three weak hydrogen bonds with the oxygen atoms of the two different hydroxyl groups of niazirin. As a result, it was revealed from Figures S3 and S4 that the interactions between ApoE4 and moringin or niazirin were significantly influenced by Asp12 and Arg15, while Asp130 had no significant contribution to the binding of these compounds.

**Figure 5:**
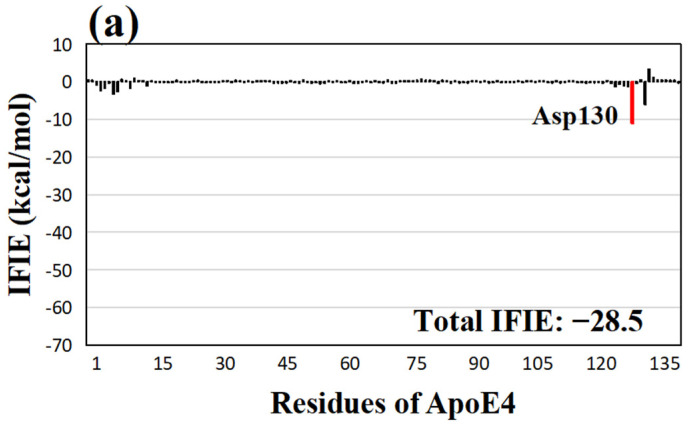

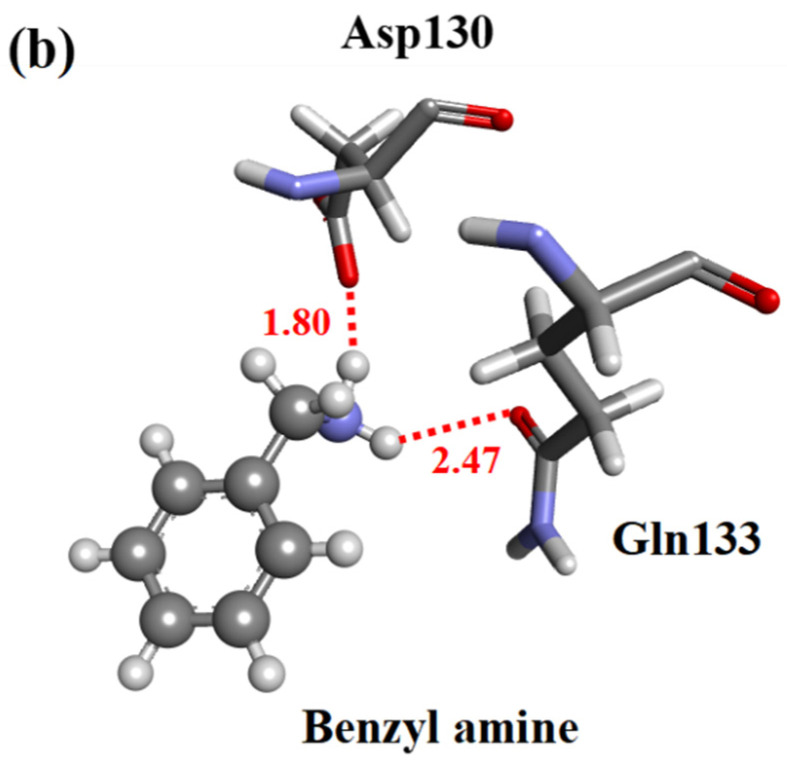
(**a**) IFIEs between the least effective compound benzyl amine and ApoE4 residues. The total IFIE of benzyl amine is also shown. The ApoE4 residue with negative IFIE greater than 10 kcal/mol is displayed with a red bar. (**b**) Structure of the interactions between benzyl amine and key ApoE4 residues. Hydrogen bonds are represented by red dotted lines, and their distances are given in Å.

As shown in Table 2, benzyl amine had the lowest total IFIE (−28.5 kcal/mol) of the 16 compounds. The total IFIE is approximately five times smaller than that of the best compound, quercetin, indicating a weak binding between benzyl amine and ApoE4. Only Asp130 of ApoE4 had a strong attractive interaction (−11 kcal/mol) with benzyl amine, as shown in Figure 5a. The hydrogen atoms of the NH_2_ group of benzyl amine formed hydrogen bonds with the oxygen atoms of Asp130 and the carbonyl oxygen of Gln133 at 1.80 and 2.47 Å, respectively, as shown in Figure 5b. Figure S5 displays the IFIE graph and interaction structure of the second weakest compound, eugenol (−33.6 kcal/mol). Only the residue Trp11 (−10.3 kcal/mol) had an IFIE greater than 10 kcal/mol, and there were no hydrogen bond interactions between ApoE4 residues and eugenol. The interaction structure and IFIE graph for the third weakest compound, vanillin, are shown in Figure S6. Only Asp130 had an IFIE higher than 10 kcal/mol, as a hydrogen bond was formed at 1.56 Å between the oxygen atom of Asp130 and the hydrogen atom of the hydroxyl group of vanillin. The IFIE graphs and interaction structures between ApoE4 residues and each of the other eight compounds are shown in Figures S7–S14, indicating how the natural compounds from *M. oleifera* and the ApoE4 residues interact with each other.

### 2.4. Classification of Compounds Using VISCANA Analysis

Using the visualized cluster analysis (VISCANA) of protein–ligand interactions [64], we classified the 26 compounds, including 16 compounds from *M. oleifera* and 10 natural compounds from cinnamon [31] based on their IFIEs with ApoE4, to elucidate the differences in their specific interactions with the ApoE4 residues. Based on the similarity of IFIEs between each compound and the ApoE4 residues existing around the ligand-binding site of ApoE4, the 26 compounds were classified into various groups, as shown in Figure S15, where red and blue colors indicate attractive and repulsive interactions, respectively, and the shading represents the magnitude of IFIE.

In our previous study [31], epicatechin from cinnamon was found to bind most strongly to ApoE4. Notably, this compound was classified into the same cluster as the most effective compound, quercetin, from *M. oleifera*, as indicated in Figure S15. The second and third effective *M. oleifera* compounds, luteolin and genistein, were also classified in the same cluster. These effective compounds were found to interact strongly with the residues Asp12 and Asp130 of ApoE4, indicating that these residues are essential for the strong interactions between ApoE4 and compounds. Notably, the same cluster included the compound daidzein, which had a rather small total IFIE (−82.7 kcal/mol) with ApoE4. Both daidzein and genistein are isoflavones and have a similar structure. Daidzein has two hydroxyl groups, while genistein has three hydroxyl groups to form strong hydrogen bonds with the aspartate residues Asp12 and Asp130, as shown in Figure 4b. In contrast, as shown in Figure S14a,b, daidzein has a smaller IFIE with Asp130 because there was no hydrogen bond formed between the hydroxyl group of daidzein and Asp130. As a result, the IFIE value between daidzein and Asp130 was significantly lower than that of genistein, leading to a smaller total IFIE for daidzein compared with that of genistein. Accordingly, strong hydrogen bonds with both the Asp12 and Asp130 residues of ApoE4 were found to be necessary for a compound to bind strongly to ApoE4.

### 2.5. Proposal of Novel Quercetin Derivatives as Potent ApoE4 Inhibitors

As quercetin was found to bind most strongly to ApoE4, we chose it as the leading compound to propose novel compounds as potent ApoE4 inhibitors. As shown in Figure 2b, the hydroxyl groups of quercetin significantly contributed to its strong interactions with the ApoE4 residues. Hence, we here considered the addition of a hydroxyl group to some different sites of quercetin to propose novel quercetin derivatives. As listed in Table 3, the hydrogen atom at each of the five introducing sites of quercetin was changed to a hydroxyl group. The proposed derivatives are defined as **Qa**, **Qb**, **Qc**, **Qd**, and **Qe**, respectively, according to the introductory site of a hydroxyl group. We used the MM method to optimize the structures of the ApoE4 complexes with these proposed derivatives, and the total IFIEs between ApoE4 and these proposed derivatives were evaluated using the ab initio FMO method. As indicated in Table 3, the total IFIEs were found to depend significantly on the hydroxylation site. **Qa**, **Qc**, and **Qe** had higher total IFIEs than the original quercetin, suggesting that the addition of a hydroxyl group to quercetin could greatly enhance the binding of these quercetin derivatives to ApoE4. Particularly, **Qe** had a total IFIE (−178.3 kcal/mol) that was 42.1 kcal/mol higher than that of quercetin (−136.2 kcal/mol), followed by **Qc** (−149.9 kcal/mol) and **Qa** (−144.1 kcal/mol). Notably, the total IFIEs of these quercetin derivatives were higher than those of our previously proposed epicatechin derivatives **Ea** (−141.6 kcal/mol) and **Ee** (−128.4 kcal/mol) [31]. Therefore, our proposed quercetin derivatives (**Qe**, **Qc**, and **Qa**) are expected to be effective ApoE4 inhibitors.

**Figure 6:**
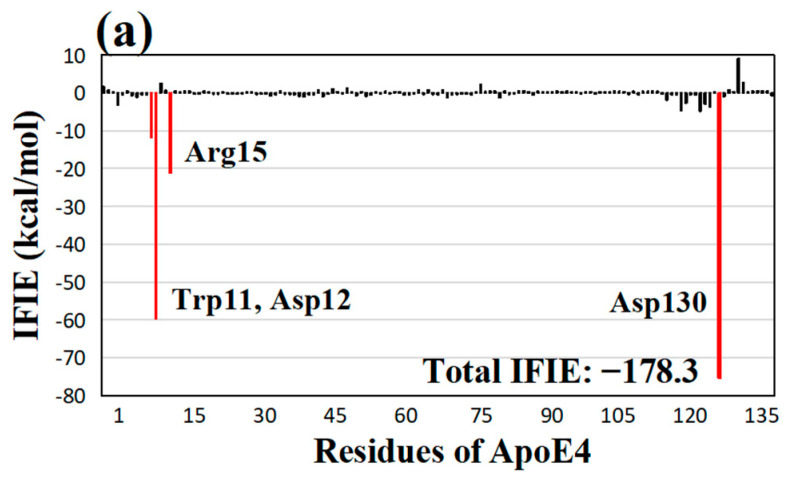

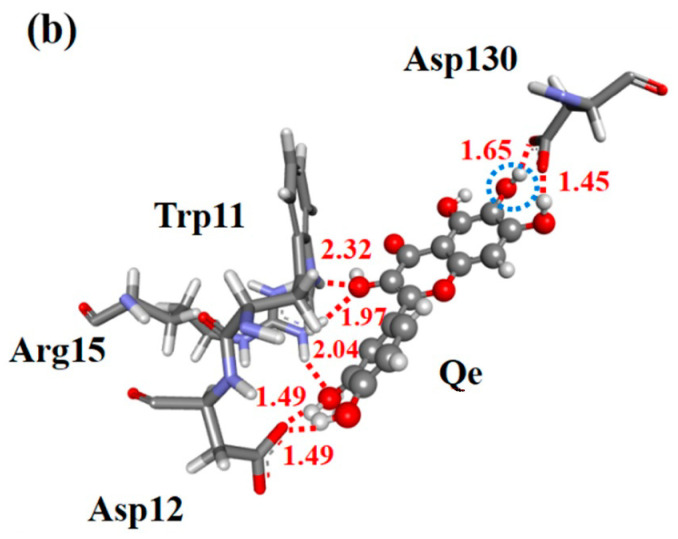
(**a**) IFIEs between the most effective quercetin derivative **Qe** and ApoE4 residues. The total IFIE of **Qe** is also shown. ApoE4 residues with negative IFIEs greater than 10 kcal/mol are displayed with red bars. (**b**) Structure of the interactions between **Qe** and key ApoE4 residues. Hydrogen bonds are represented by red dotted lines, and their distances are given in Å. The introduced OH group at the **e**-site of quercetin is shown in a blue dotted circle.

To elucidate the reason for this notable improvement in total IFIE by the hydroxylation, we investigated the IFIEs between **Qa**/**Qc**/**Qe** and the ApoE4 residues. Figure 6a shows the results for the **Qe** derivative, indicating that Trp11 (−11.8 kcal/mol), Asp12 (−59.6 kcal/mol), Arg15 (−21.1 kcal/mol), and Asp130 (−75.3 kcal/mol) exhibited higher IFIEs with the best quercetin derivative, **Qe**. Trp11 and Asp12 showed remarkably similar IFIEs with quercetin (Figure 2a). In contrast, Arg15 and Asp130 had higher IFIEs with **Qe** compared with those of quercetin. In fact, the addition of a hydroxyl group to the **e**-site of quercetin increased the IFIE between Asp130 and quercetin by 33.9 kcal/mol, as seen in Figure 2a and Figure 6a. A similar increase of 5.7 kcal/mol was seen in the IFIE between Arg15 and **Qe**. As a result, **Qe** had the highest total IFIE of −178.3 kcal/mol among our proposed quercetin derivatives. To reveal the reason for this enhancement, we investigated the interaction structure between **Qe** and some key ApoE4 residues. Figure 6b demonstrates that the hydrogen atom of the newly added hydroxyl group at the **e**-site of quercetin formed a strong hydrogen bond with the oxygen atom of Asp130 at 1.65 Å, leading to an increased IFIE between **Qe** and Asp130 than that for quercetin. In addition, the residues Trp11, Asp12, and Arg15 contributed to hydrogen bonds with **Qe**.

The IFIEs between the quercetin derivative **Qc** and ApoE4 residues are shown in Figure S16a. Trp11 (−11.2 kcal/mol), Asp12 (−69.0 kcal/mol), Arg15 (−12.1 kcal/mol), and Asp130 (−44.3 kcal/mol) displayed higher IFIEs than 10 kcal/mol. The IFIEs of Asp12 and Asp130 were higher for **Qc** as compared with those for the ApoE4−quercetin complex. The newly added hydroxyl group at the **c**-site of quercetin formed a hydrogen bond with Arg15 at 2.09 Å, as shown in Figure S16b. Figure S17a indicates that **Qa** had strong attractive interactions with Asp12 (−58.9 kcal/mol), Arg15 (−17.2 kcal/mol), and Asp130 (−46.1 kcal/mol). The IFIEs of Arg15 and Asp130 residues slightly increased compared with the ApoE4−quercetin complex. As a result, the total IFIE of **Qa** is higher than quercetin. However, as seen in Figure S17b, the introduced hydroxyl group at the **a**-site failed to form hydrogen bonds with ApoE4 residues, resulting in a similar total IFIE of **Qa** compared with the pristine quercetin. The results for **Qb** are shown in Figure S18a, indicating the higher IFIEs of Trp11 (−11.3 kcal/mol), Asp12 (−60.8 kcal/mol), Arg15 (−10.0 kcal/mol), and Asp130 (−42.4 kcal/mol). Compared with the IFIEs for the ApoE4−quercetin complex, the IFIEs of Asp12 and Arg15 slightly decreased. As a result, **Qb** has a lower total IFIE than quercetin. Additionally, as illustrated in Figure S18b, the added hydroxyl group at the **b**-site did not form a hydrogen bond with ApoE4 residues, indicating that the hydroxylation at the **b**-site of quercetin is not suitable for enhancing the binding between quercetin and ApoE4.

**Figure 7:**
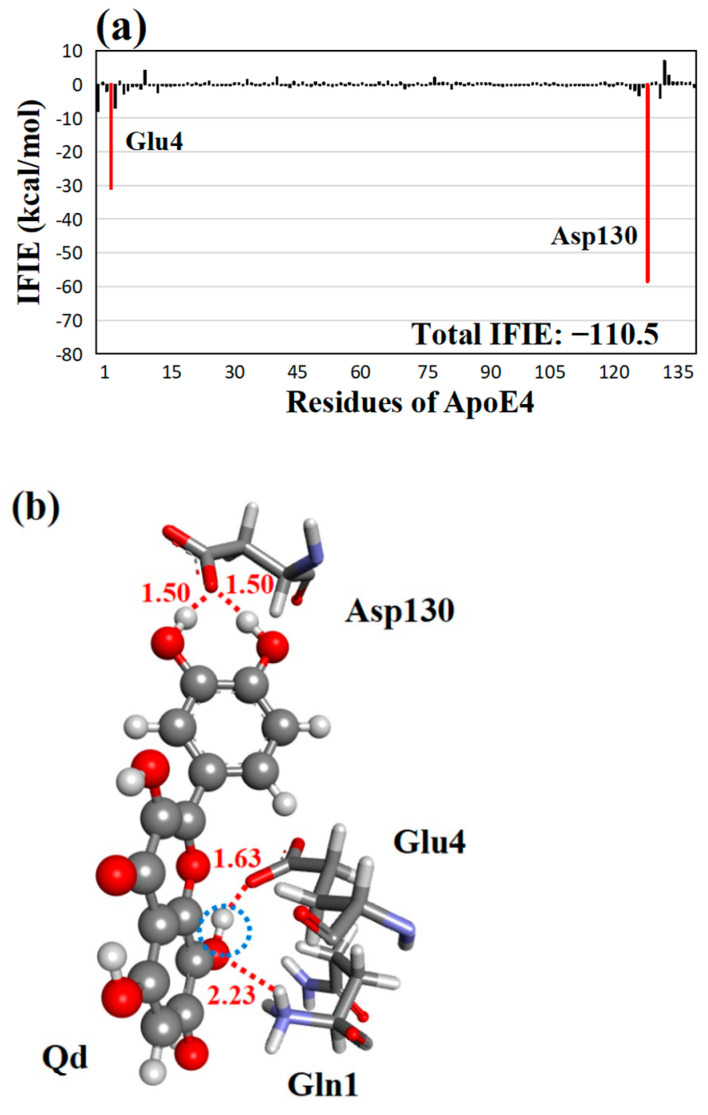
(**a**) IFIEs between the least effective quercetin derivative, **Qd**, and ApoE4 residues. The total IFIE of **Qd** is also shown. ApoE4 residues with negative IFIEs greater than 10 kcal/mol are displayed with red bars. (**b**) Structure of the interactions between **Qd** and key ApoE4 residues. Hydrogen bonds are represented by red dotted lines, and their distances are given in Å. The introduced OH group at the **d**-site of quercetin is shown in a blue dotted circle.

Among the five suggested quercetin derivatives, **Qd** had the lowest total IFIE (−110.5 kcal/mol), as shown in Table 3. To determine the cause of this lower IFIE, we compared the IFIEs for the best derivative **Qe** (−178.3 kcal/mol) and **Qd**. As shown in Figure 7a, **Qd** had strong attractive interactions with only Glu4 (−31.0 kcal/mol) and Asp130 (−58.6 kcal/mol). On the other hand, **Qe** had a higher IFIE (−75.3 kcal/mol) with Asp130 (Figure 6a). In addition, **Qe** had strong interactions with Trp11, Asp12, and Arg15 residues. In the ApoE4−**Qd** complex (Figure 7b), these residues were not involved in the interaction with **Qd**. As a result, the total IFIE of **Qd** was significantly smaller than that of **Qe**. Therefore, it is concluded that the binding affinity of quercetin derivatives to ApoE4 is significantly affected by the hydroxylation site of quercetin and that the site should be considered for forming new hydrogen bonds with ApoE4 residues. Notably, the present study was a computational analysis, and experiments should be conducted to confirm these findings.

## 3. Methods of Molecular Simulations

To accurately analyze the interactions between ApoE4 and the compounds, we utilized the ab initio FMO approach [32]. This technique involves dividing the target protein into smaller fragments and assessing the electronic states of individual fragments and fragment pairs while considering the electrostatic potentials of neighboring fragments. Subsequently, the electronic states of the target protein are determined based on the electronic states of the fragments and fragment pairs. Using the FMO analysis, we can derive the interaction energies between the fragments, which are referred to as inter-fragment interaction energies (IFIEs) [65]. These IFIEs are valuable for investigating the specific interactions between the desired protein residues and their biological inhibitors.

The current investigation started with a selection of compounds found in various parts of *M. oleifera* [11]. Based on the neuroprotective effects of these phytochemicals, we selected 25 compounds for our present investigation. The selected 25 compounds were evaluated according to Lipinski’s rule of five [34], which is considered a key criterion for pharmacologically active molecules. The web tool SwissADME [33] was used to analyze the chemical characteristics of the compounds. Of the 25 compounds, only 16 compounds met Lipinski’s criterion and were selected as candidates for the ApoE4 inhibitor. The B3LYP/6-31G (d, p) approach in the ab initio molecular orbital calculation program Gaussian16 [35] was utilized to fully optimize their three-dimensional (3D) structures, which were retrieved from the PubChem database [66]. The HF/6-31G(d) method and the RESP analysis [36] of Gaussian16 [35] were used to determine the charge distributions of the optimized structures. These RESP charges were used in the MM force fields for the compounds in the MM optimizations and the protein–ligand docking simulations.

The three-dimensional structure of ApoE4 [67] was taken from the protein data bank [68] (PDB ID: 6NCO) and docked all compounds into the binding site using AutoDock 4.2.6 [37], a protein–ligand docking simulation tool. In the docking simulations, the grid box size was set to 19 × 19 × 19 Å^3^. This was around 1.5 times the size of the crystallized ligand in the co-crystallized complex [67]. The grid box’s center is situated at the center of the ligand. There were 256 candidate poses, and the distance between each one was designated as 1.5 Å for clustering. The maximum energy evaluations (ga_num_evals) allowed for each docking run were determined to be 2,500,000. AutoDock 4.2.6 [37] produced several clusters, but we picked the one with the most poses as a representative structure and utilized it in the following MM and FMO simulations.

In explicit water, the candidate structures for ApoE4 complexes with the compound were thoroughly analyzed using classical MM and the molecular dynamics simulation program AMBER18 [41]. The stability of protein–ligand complexes in water was achieved by solvating water molecules within 8 Å from the surface of the complex in the MM optimizations. For this, we used the solvateShell command of AMBER18 [41]. The other atoms were fixed to optimize the positions of the added water molecules. After that, the positions of all atoms of protein, ligand, and water molecules were completely optimized. To represent the ApoE4 residues, the water molecules, and the compounds, the MM optimizations utilized the AMBER FF99-SBLIN force field [38], the TIP3P model [39], and the general AMBER force field [40].

The precise investigation of MM-optimized complexes was performed with ab initio FMO calculations [32] to clarify their binding properties. The FMO results helped us assess the binding affinity between ApoE4 and the compounds and determine the compound that binds ApoE4 most efficiently. Each ApoE4 residue, compound, and water molecule was classified as distinct fragments in the FMO calculations. We analyzed the interaction between the androgen receptor and its ligands using MP2/6-31G(d) calculations in our earlier research [69]. The binding energies of the receptor and its ligands were found to be correlated with the binding energies determined from the results. Therefore, we used the MP2/6-31G(d) method within the ABINIT-MPVer6.0 [70] tool to accurately assess how ApoE4 and compounds interact. We were able to identify the ApoE4 residues that play a key role in their interactions by analyzing the IFIEs from ab initio FMO calculations.

## 4. Conclusions

To investigate the specific interactions between ApoE4 and natural compounds contained in *M. oleifera* and propose novel potent inhibitors against ApoE4, we used molecular docking, classical MM, and ab initio FMO simulations. According to the FMO results, quercetin was found to bind most strongly to ApoE4 and be a promising candidate compound as an efficient ApoE4 inhibitor. We furthermore proposed five quercetin derivatives by introducing a hydroxyl group and investigated their binding properties to ApoE4. The results demonstrated that the addition of a hydroxyl group to some sites of quercetin significantly enhances the interactions between quercetin and ApoE4. Particularly, the compound **Qe** (Table 3) has strong interactions with ApoE4 residues and is expected to be a potent inhibitor against ApoE4. The FMO results also revealed that the Trp11, Asp12, Arg15, and Asp130 residues of ApoE4 mainly contribute to the strong binding between ApoE4 and the quercetin derivatives. The present findings obtained using ab initio FMO calculations will be helpful for the development of effective ApoE4 inhibitors, which will result in the successful treatment of AD.

## Figures and Tables

**Table 1:** The cluster ranking, lowest binding energy (BE: kcal/mol), and number of poses for the selected cluster obtained with the AutoDock 4.2.6 program [37]. The 256 poses were created and clustered based on their structural similarity, and each cluster was ranked in the order of lowest BE between ApoE4 and each compound. We selected the cluster with the highest number of poses because there is a high probability that the compound has one of the conformations in the cluster. This cluster was used as a potential candidate for the complex structure in the subsequent molecular simulations.

Compound	Cluster Rank	BE (kcal/mol)	Poses
Crystallized ligand	2	−5.97	165
(**1**) Benzyl amine	1	−2.60	212
(**2**) Vanillin	4	−2.17	86
(**3**) Eugenol	2	−2.64	159
(**4**) Gallic acid	1	−2.83	133
(**5**) Syringic acid	1	−2.70	106
(**6**) Ferulic acid	5	−2.30	52
(**7**) Sinapic acid	1	−3.43	90
(**8**) Niazirin	4	−3.53	90
(**9**) Moringin	1	−4.68	70
(**10**) Daidzein	1	−4.13	163
(**11**) Genistein	1	−4.77	78
(**12**) Apigenin	1	−3.68	91
(**13**) Kaempferol	3	−3.54	67
(**14**) Luteolin	3	−3.86	39
(**15**) Quercetin	1	−4.02	43
(**16**) Aurantiamide acetate	14	−2.31	19

**Table 2:** ApoE4 residues involved in hydrogen bond interactions with each of the compounds, and total inter-fragment interaction energies (IFIE: kcal/mol) between each compound and all AApoE4 residues calculated using the ab initio FMO method [32].

Compounds	Residues Involved in H Bonds	Total IFIE
(**1**) Benzyl amine	Asp130, Gln133	−28.5
(**2**) Vanillin	Asp130	−45.7
(**3**) Eugenol	No residue	−33.6
(**4**) Gallic acid	Asp130	−82.6
(**5**) Syringic acid	Glu4, Gly8, Gln133	−66.1
(**6**) Ferulic acid	Gln1, Glu4, Asp12	−51.6
(**7**) Sinapic acid	Glu4, Asp12, Arg15	−80.6
(**8**) Niazirin	Asp12, Arg15	−101.5
(**9**) Moringin	Asp12, Arg15	−102.7
(**10**) Daidzein	Asp12, Ala129	−82.7
(**11**) Genistein	Asp12, Ala129, Asp130	−109.1
(**12**) Apigenin	Glu4, Arg15, Asp130	−73.6
(**13**) Kaempferol	Glu4, Arg15, Asp130	−76.1
(**14**) Luteolin	Asp12, Arg15, Asp130	−109.5
(**15**) Quercetin	Trp11, Asp12, Arg15, Asp130	−136.2
(**16**) Aurantiamide acetate	No residue	−51.5

**Table 3:** ApoE4 residues involved in hydrogen bond interactions with each of the quercetin derivatives, and total IFIEs (kcal/mol) between all ApoE4 residues and quercetin or its derivatives proposed in our present study. Our proposed derivatives are defined as the compounds **Qa**–**Qe** depending on the site replaced with a hydroxyl group. For example, in the compound **Qa**, the hydrogen atom at the **a**-site of quercetin shown in the figure is replaced with a hydroxyl group.

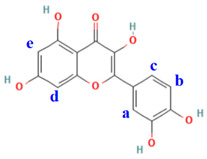		
Compounds	Residues Involved in H Bonds	Total IFIE
**Quercetin**	Trp11, Asp12, Arg15, Asp130	−136.2
**Qa**	Trp11, Asp12, Arg15, Asp130	−144.1
**Qb**	Trp11, Asp12, Arg15, Asp130	−133.9
**Qc**	Asp12, Arg15, Asp130	−149.9
**Qd**	Gln1, Glu4, Asp130	−110.5
**Qe**	Trp11, Asp12, Arg15, Asp130	−178.3

## Data Availability

Data are contained within the article and supplementary materials.

## Supplementary Materials

The following supporting information can be downloaded at: https://www.mdpi.com/article/10.3390/molecules28248035/s1, Figure S1: (a) Surface representation of the optimized ApoE4 structure. The binding cavity for its ligand is shown by the red dotted circle. (b) MM optimized structure of the ApoE4−quercetin complex.; Figure S2: The docked (blue) and the crystallized (red) conformations of the native ligand in the ligand-binding site of ApoE4.; Figure S3: (a) IFIEs between the compound moringin and ApoE4 residues. The total IFIE of moringin is also shown. ApoE4 residues with negative IFIEs greater than 10 kcal/mol are displayed with red bars. (b) Structure of the interactions between moringin and key ApoE4 residues. The hydrogen bonds are shown as red dotted lines and their distances are given in Å.; Figure S4: (a) IFIEs between the compound niazirin and ApoE4 residues. The total IFIE of niazirin is also shown. ApoE4 residues with negative IFIEs greater than 10 kcal/mol are displayed with red bars. (b) Structure of the interactions between niazirin and key ApoE4 residues. The hydrogen bonds are shown as red dotted lines and their distances are given in Å.; Figure S5: (a) IFIEs between the compound eugenol and ApoE4 residues. The total IFIE of eugenol is also shown. ApoE4 residue with negative IFIE greater than 10 kcal/mol is displayed with a red bar. (b) Structure of the interactions between eugenol and surrounding ApoE4 residues. There is no hydrogen bond between ApoE4 residues and eugenol.; Figure S6: (a) IFIEs between the compound vanillin and ApoE4 residues. The total IFIE of vanillin is also shown. ApoE4 residue with negative IFIE greater than 10 kcal/mol is displayed with a red bar. (b) Structure of the interactions between vanillin and key ApoE4 residues. The hydrogen bond is shown as a red dotted line and its distance is given in Å.; Figure S7: (a) IFIEs between the compound aurantiamide acetate and ApoE4 residues. The total IFIE of aurantiamide acetate is also shown. ApoE4 residues with negative IFIEs greater than 10 kcal/mol are displayed with red bars. (b) Structure of the interactions between aurantiamide acetate and surrounding ApoE4 residues. There is no hydrogen bond between aurantiamide acetate and ApoE4 residues.; Figure S8: (a) IFIEs between the compound ferulic acid and ApoE4 residues. The total IFIE of ferulic acid is also shown. ApoE4 residues with negative IFIEs greater than 10 kcal/mol are displayed with red bars. (b) Structure of the interactions between ferulic acid and key ApoE4 residues. The hydrogen bonds are shown as red dotted lines and their distances are given in Å.; Figure S9: (a) IFIEs between the compound syringic acid and ApoE4 residues. The total IFIE of syringic acid is also shown. ApoE4 residues with negative IFIEs greater than 10 kcal/mol are displayed with red bars. (b) Structure of the interactions between syringic acid and key ApoE4 residues. The hydrogen bonds are shown as red dotted lines and their distances are given in Å.; Figure S10: (a) IFIEs between the compound apigenin and ApoE4 residues. The total IFIE of apigenin is also shown. ApoE4 residues with negative IFIEs greater than 10 kcal/mol are displayed with red bars. (b) Structure of the interactions between apigenin and key ApoE4 residues. The hydrogen bonds are shown as red dotted lines and their distances are given in Å.; Figure S11: (a) IFIEs between the compound kaempferol and ApoE4 residues. The total IFIE of kaempferol is also shown. ApoE4 residues with negative IFIEs greater than 10 kcal/mol are displayed with red bars. (b) Structure of the interactions between kaempferol and key ApoE4 residues. The hydrogen bonds are shown as red dotted lines and their distances are given in Å.; Figure S12: (a) IFIEs between the compound sinapic acid and ApoE4 residues. The total IFIE of sinapic acid is also shown. ApoE4 residues with negative IFIEs greater than 10 kcal/mol are displayed with red bars. (b) Structure of the interactions between sinapic acid and key ApoE4 residues. The hydrogen bonds are shown as red dotted lines and their distances are given in Å.; Figure S13: (a) IFIEs between the compound gallic acid and ApoE4 residues. The total IFIE of gallic acid is also shown. ApoE4 residue with negative IFIE greater than 10 kcal/mol is displayed with a red bar. (b) Structure of the interactions between gallic acid and key ApoE4 residues. The hydrogen bonds are shown as red dotted lines and their distances are given in Å.; Figure S14: (a) IFIEs between the compound daidzein and ApoE4 residues. The total IFIE of daidzein is also shown. ApoE4 residues with negative IFIEs greater than 10 kcal/mol are displayed with red bars. (b) Structure of the interactions between daidzein and key ApoE4 residues. The hydrogen bonds are shown as red dotted lines and their distances are given in Å.; Figure S15: Classification of 26 natural compounds using visualized cluster analysis (VISCANA) based on protein-ligand interactions [69]. Each compound is indicated on the vertical axis. The horizontal axis on the right represents the ApoE4 residues existing within 10 Å of the compound. The red and blue colors indicate attractive and repulsive interactions, respectively, and the shading represents the magnitude of IFIE. The tree on the left represents the clustering of compounds based on their IFIEs with ApoE4 residues.; Figure S16: (a) IFIEs between the quercetin derivative **Qc** and ApoE4 residues. The total IFIE of **Qc** is also shown. ApoE4 residues with negative IFIEs greater than 10 kcal/mol are displayed with red bars. (b) Structure of the interactions between **Qc** and key ApoE4 residues. Hydrogen bonds are represented by red dotted lines and their distances are given in Å. The introduced hydroxyl group at the **c**-site of quercetin is shown in a blue dotted circle.; Figure S17: (a) IFIEs between the quercetin derivative **Qa** and ApoE4 residues. The total IFIE of **Qa** is also shown. ApoE4 residues with negative IFIEs greater than 10 kcal/mol are displayed with red bars. (b) Structure of the interactions between **Qa** and key ApoE4 residues. Hydrogen bonds are represented by red dotted lines and their distances are given in Å. The introduced hydroxyl group at the **a**-site of quercetin is shown in a blue dotted circle.; Figure S18: (a) IFIEs between the quercetin derivative **Qb** and ApoE4 residues. The total IFIE of **Qb** is also shown. ApoE4 residues with negative IFIEs greater than 10 kcal/mol are displayed with red bars. (b) Structure of the interactions between **Qb** and key ApoE4 residues. Hydrogen bonds are represented by red dotted lines and their distances are given in Å. The introduced hydroxyl group at the **b**-site of quercetin is shown in a blue dotted circle.; Table S1: Chemical properties of the 16 compounds contained in *Moringa oleifera* [11]. Their PubChem ID, molecular weight (MW), number of rotatable bonds (RB), number of H-bond acceptors (HBA), number of H-bond donors (HBD), octanol-water partition coefficient (LogP), and total polar surface area (TPSA) were computed using SwissADME web tool [35]. The number of rotatable bonds (RB) is counted by omitting the bonds connected to the hydrogen atoms.
