# Supplementary material for: In Silico Design of Natural Inhibitors of ApoE4 from the Plant Moringa oleifera: Molecular Docking and Ab Initio Fragment Molecular Orbital Calculations
Source: Molecules. 2023 Dec 11;28(24):8035. doi: 10.3390/molecules28248035 (PMC10745539; doi:10.3390/molecules28248035)
Supplement: Supplementary file 1 [file molecules-28-08035-s001.zip › molecules-2745674-supplementary.pdf]

Supporting information of

In silico design of natural inhibitors against apolipoprotein E4 from plant *Moringa Oleifera*: Molecular docking and *ab initi*o fragment molecular orbital calculations

Divya Shaji, Yoshinobu Nagura, Haruna Sabishiro, Ryo Suzuki,
and Noriyuki Kurita *

Department of Computer Science and Engineering, Toyohashi University of Technology, Tempaku-cho, Toyohashi, Aichi, 441-8580, Japan

***Corresponding author**
Noriyuki Kurita, Associate professor, Ph.D.
Department of Computer Science and Engineering
Toyohashi University of Technology
Tempaku-cho, Toyohashi, Aichi, 441-8580, Japan

Figure S1: (a) Surface representation of the optimized ApoE4 structure. The binding cavity for its ligand is shown by the red dotted circle. (b) MM optimized structure of the ApoE4−quercetin complex.

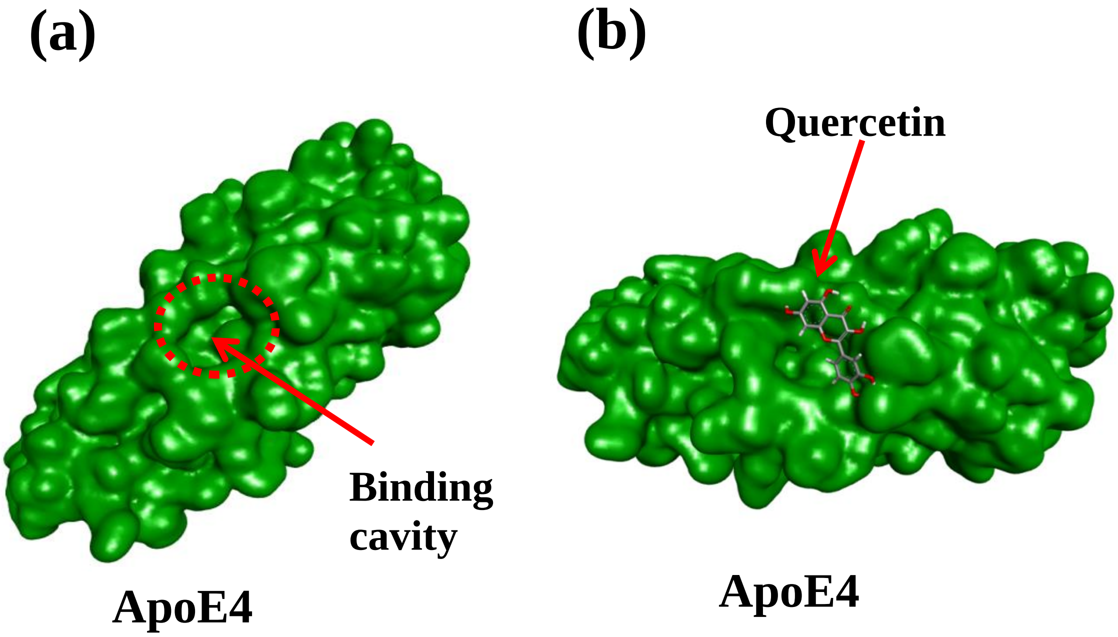

Figure S2: The docked (blue) and the crystallized (red) conformations of the native ligand in the ligand-binding site of ApoE4.

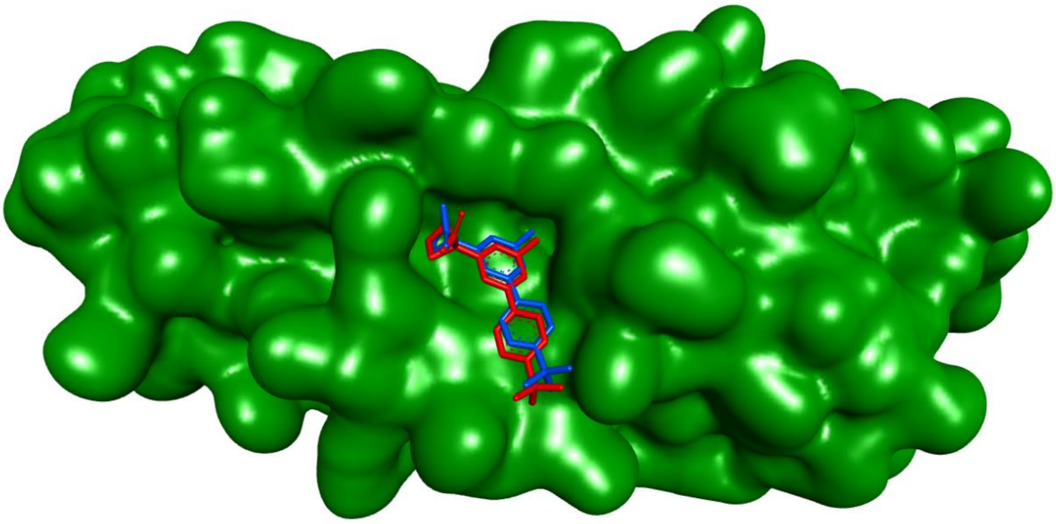

ApoE4

Figure S3: (a) IFIEs between the compound moringin and ApoE4 residues. The total IFIE of moringin is also shown. ApoE4 residues with negative IFIEs greater than 10 kcal/mol are displayed with red bars. **(b)** Structure of the interactions between moringin and key ApoE4 residues. The hydrogen bonds are shown as red dotted lines and their distances are given in Å.

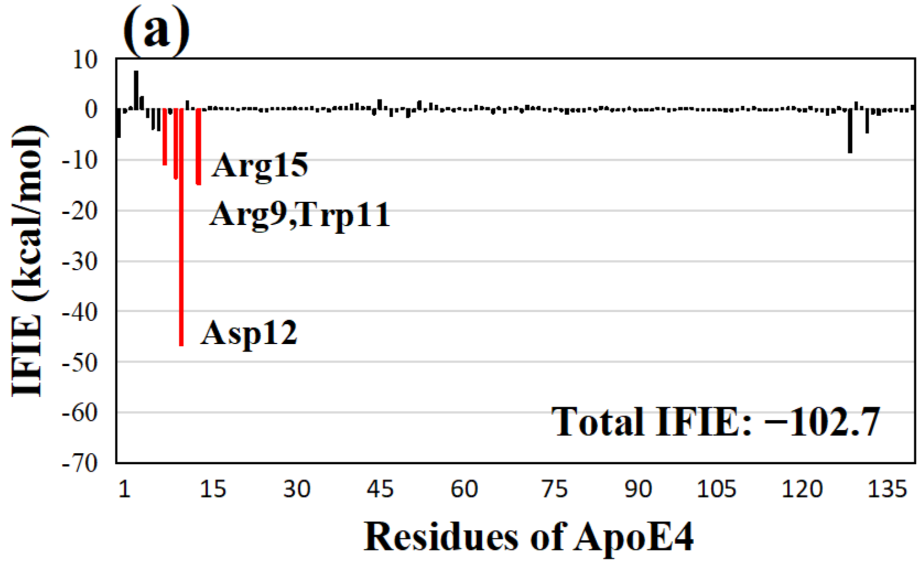

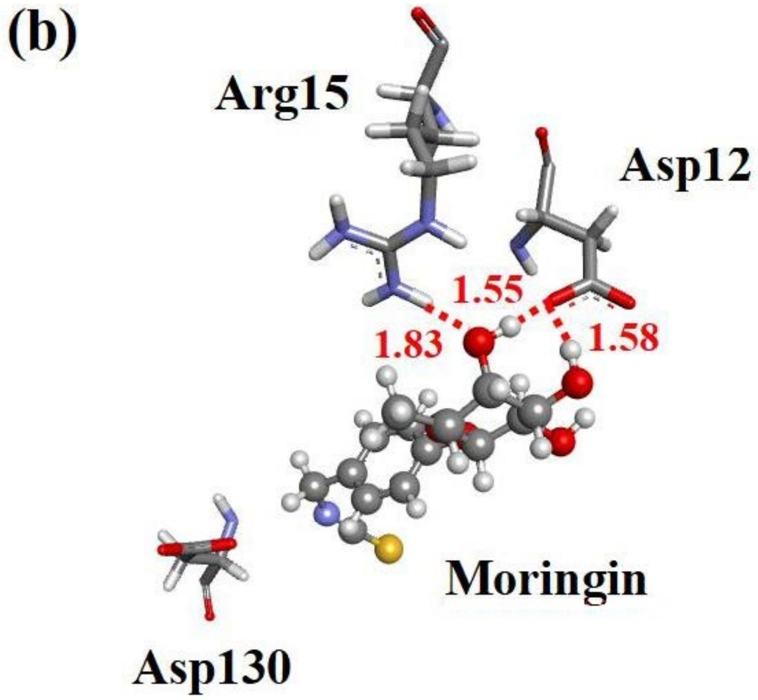

Figure S4: (a) IFIEs between the compound niazirin and ApoE4 residues. The total IFIE of niazirin is also shown. ApoE4 residues with negative IFIEs greater than 10 kcal/mol are displayed with red bars. **(b)** Structure of the interactions between niazirin and key ApoE4 residues. The hydrogen bonds are shown as red dotted lines and their distances are given in Å.

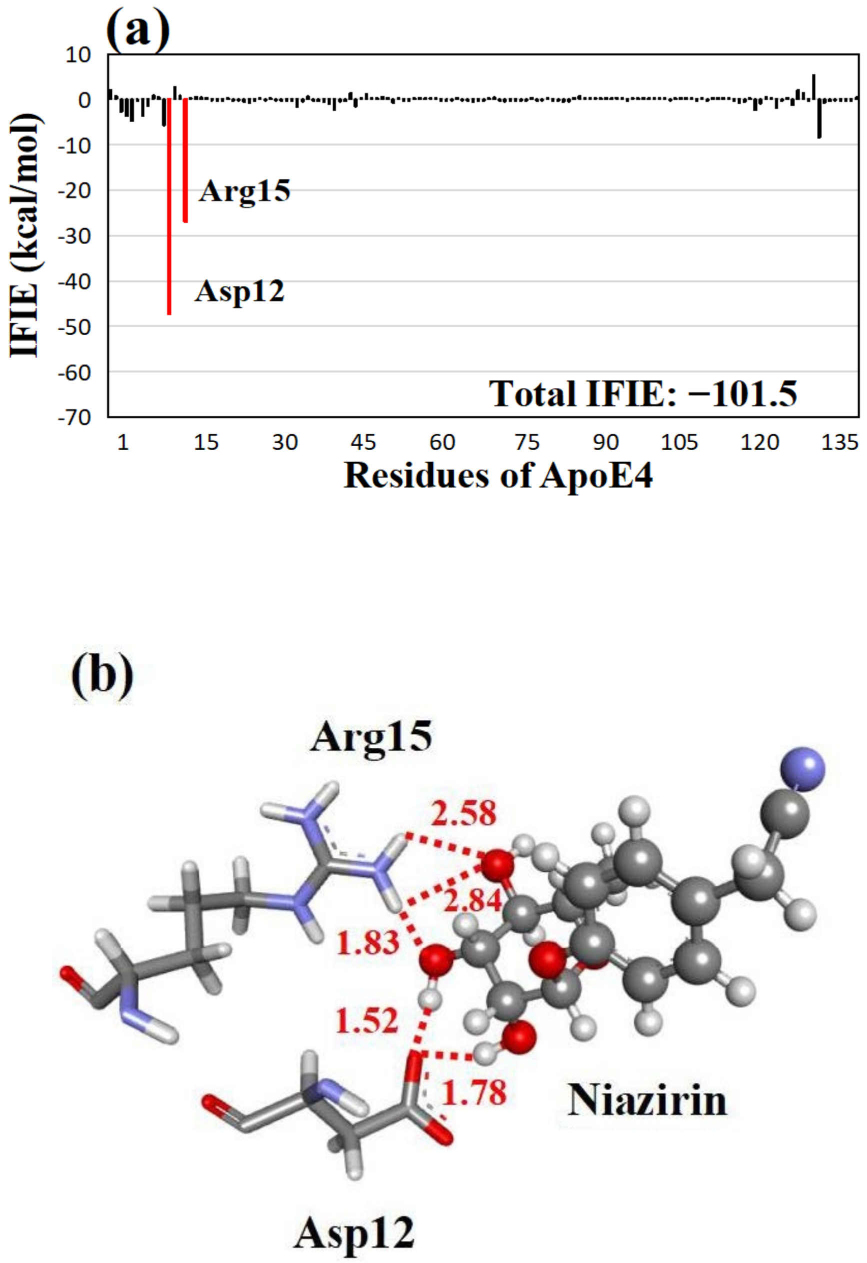

Figure S5: (a) IFIEs between the compound eugenol and ApoE4 residues. The total IFIE of eugenol is also shown. ApoE4 residue with negative IFIE greater than 10 kcal/mol is displayed with a red bar. **(b)** Structure of the interactions between eugenol and surrounding ApoE4 residues. There is no hydrogen bond between ApoE4 residues and eugenol.

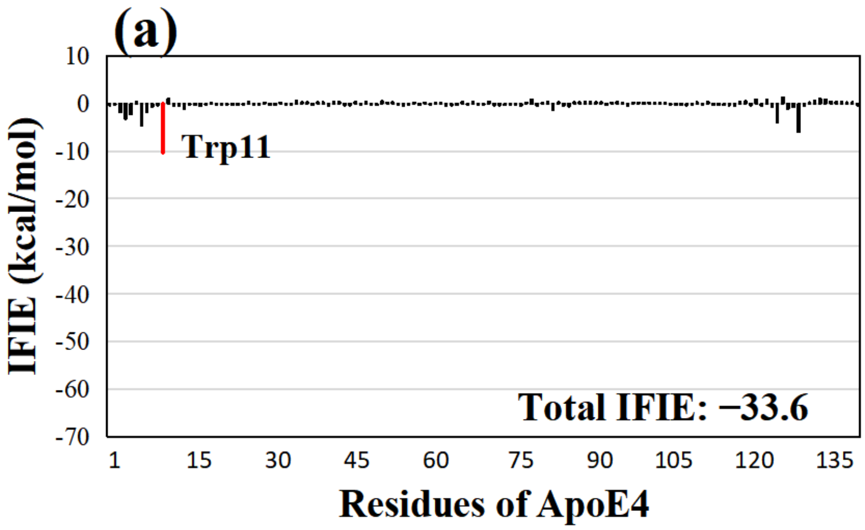

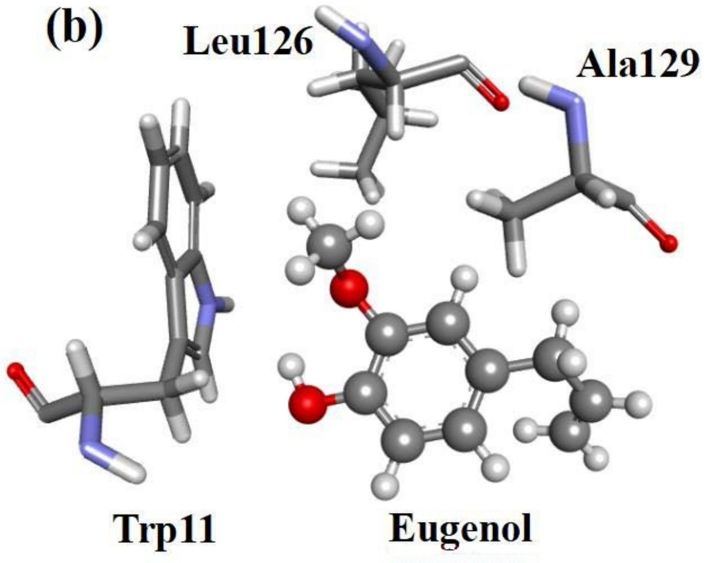

Figure S6: (a) IFIEs between the compound vanillin and ApoE4 residues. The total IFIE of vanillin is also shown. ApoE4 residue with negative IFIE greater than 10 kcal/mol is displayed with a red bar. **(b)** Structure of the interactions between vanillin and key ApoE4 residues. The hydrogen bond is shown as a red dotted line and its distance is given in Å.

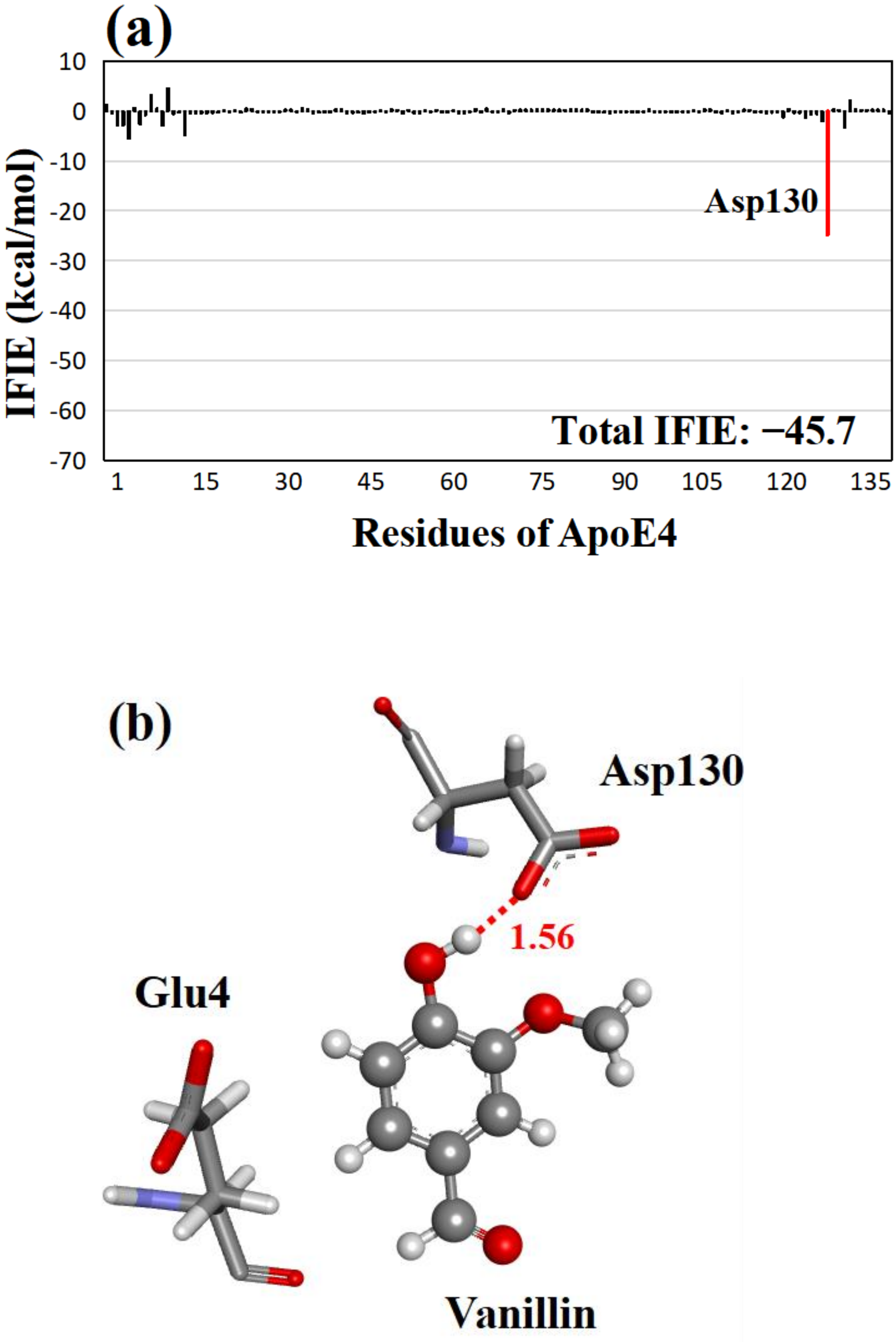

Figure S7: (a) IFIEs between the compound aurantiamide acetate and ApoE4 residues. The total IFIE of aurantiamide acetate is also shown. ApoE4 residues with negative IFIEs greater than 10 kcal/mol are displayed with red bars. **(b)** Structure of the interactions between aurantiamide acetate and surrounding ApoE4 residues. There is no hydrogen bond between aurantiamide acetate and ApoE4 residues.

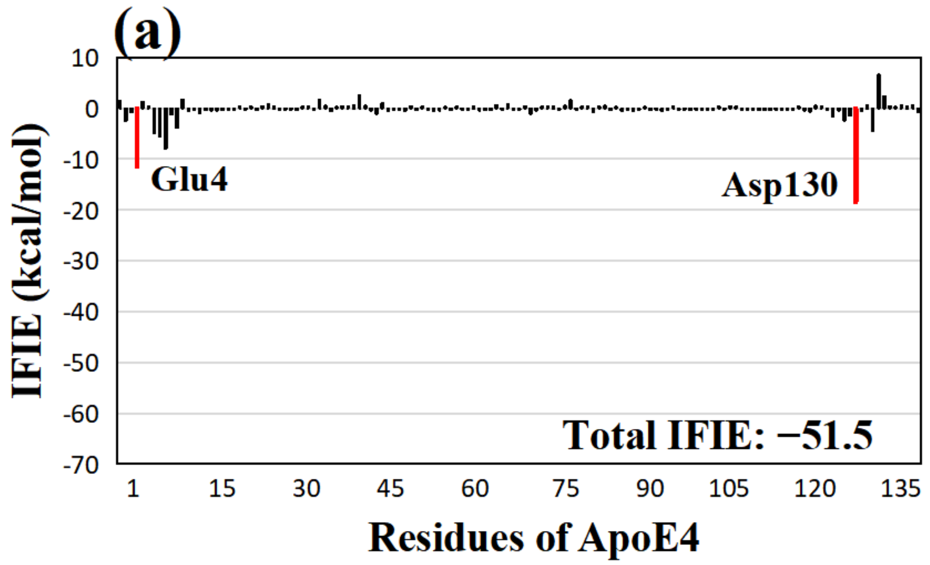

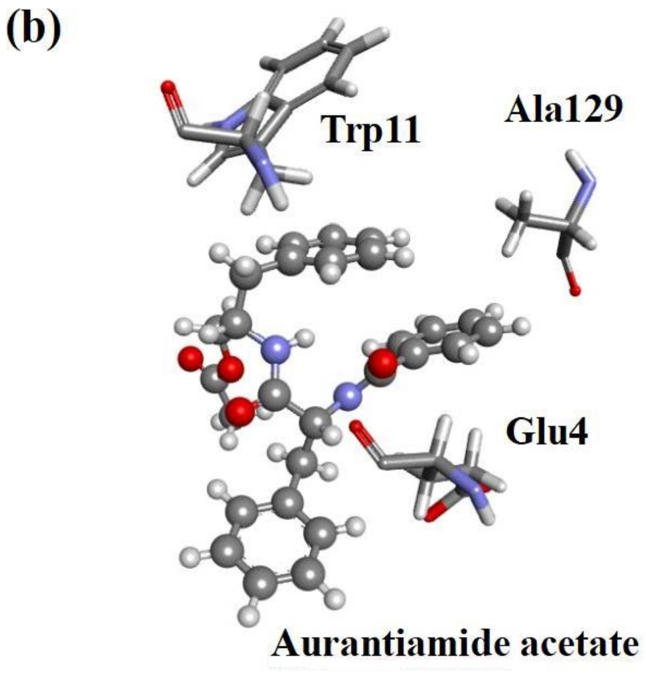

Figure S8: (a) IFIEs between the compound ferulic acid and ApoE4 residues. The total IFIE of ferulic acid is also shown. ApoE4 residues with negative IFIEs greater than 10 kcal/mol are displayed with red bars. **(b)** Structure of the interactions between ferulic acid and key ApoE4 residues. The hydrogen bonds are shown as red dotted lines and their distances are given in Å.

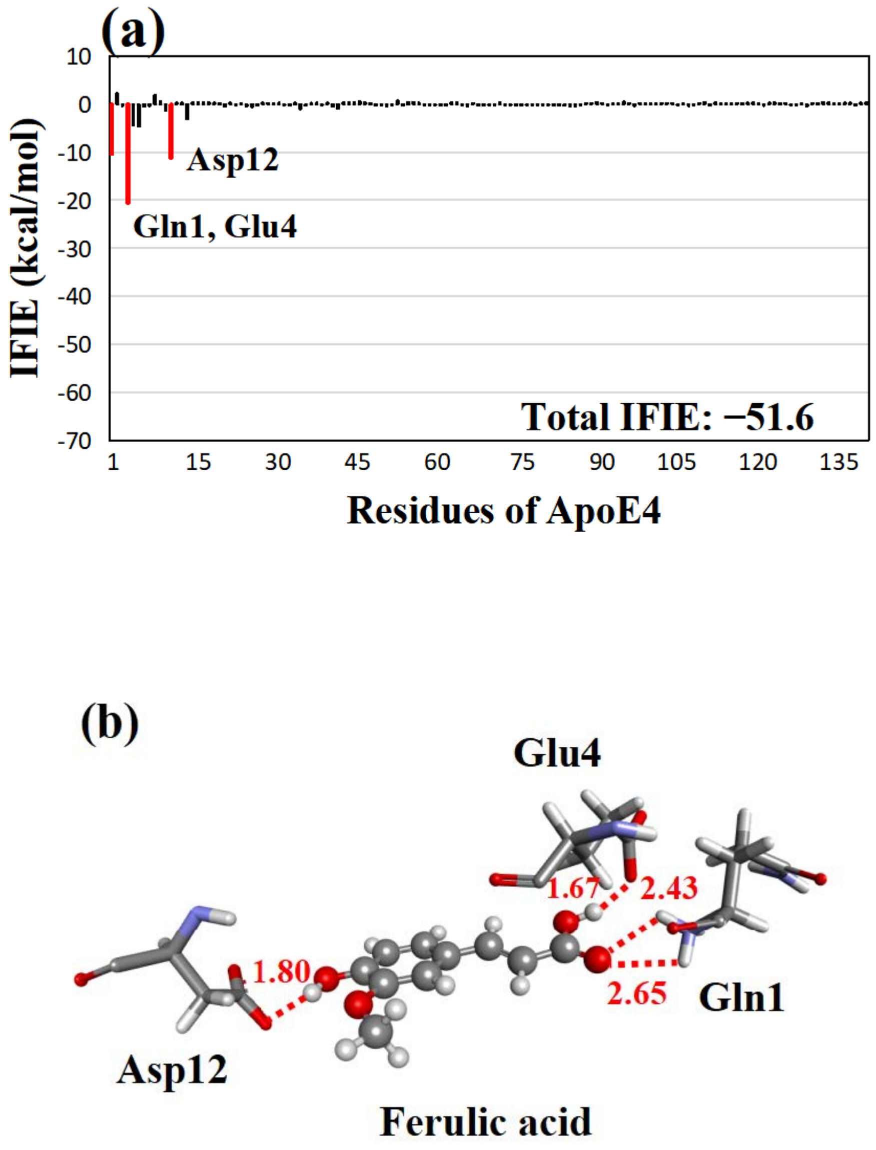

Figure S9: (a) IFIEs between the compound syringic acid and ApoE4 residues. The total IFIE of syringic acid is also shown. ApoE4 residues with negative IFIEs greater than 10 kcal/mol are displayed with red bars. **(b)** Structure of the interactions between syringic acid and key ApoE4 residues. The hydrogen bonds are shown as red dotted lines and their distances are given in Å.

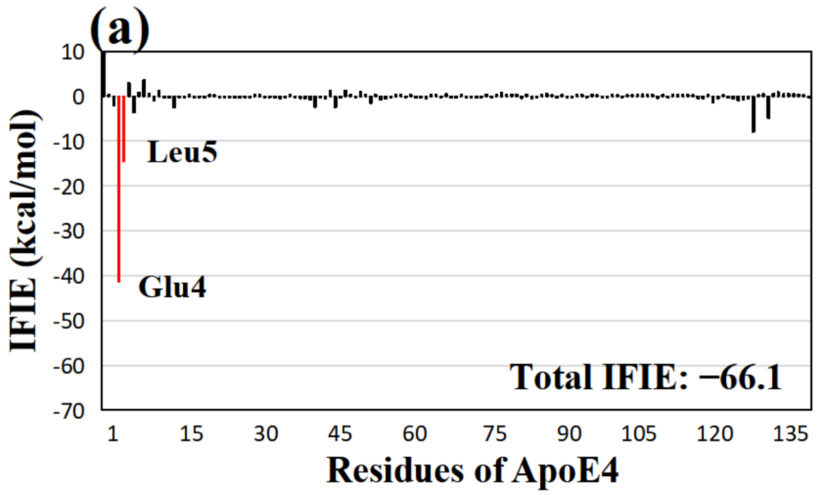

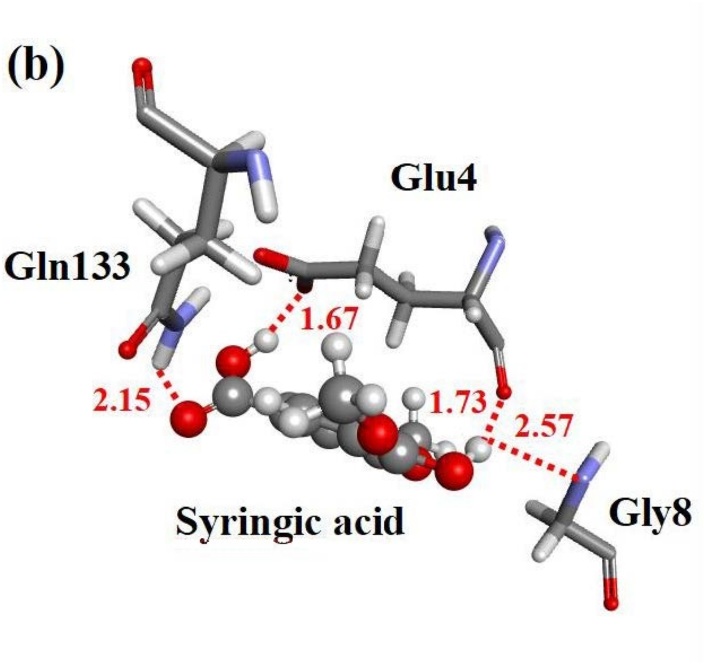

Figure S10: (a) IFIEs between the compound apigenin and ApoE4 residues. The total IFIE of apigenin is also shown. ApoE4 residues with negative IFIEs greater than 10 kcal/mol are displayed with red bars. **(b)** Structure of the interactions between apigenin and key ApoE4 residues. The hydrogen bonds are shown as red dotted lines and their distances are given in Å.

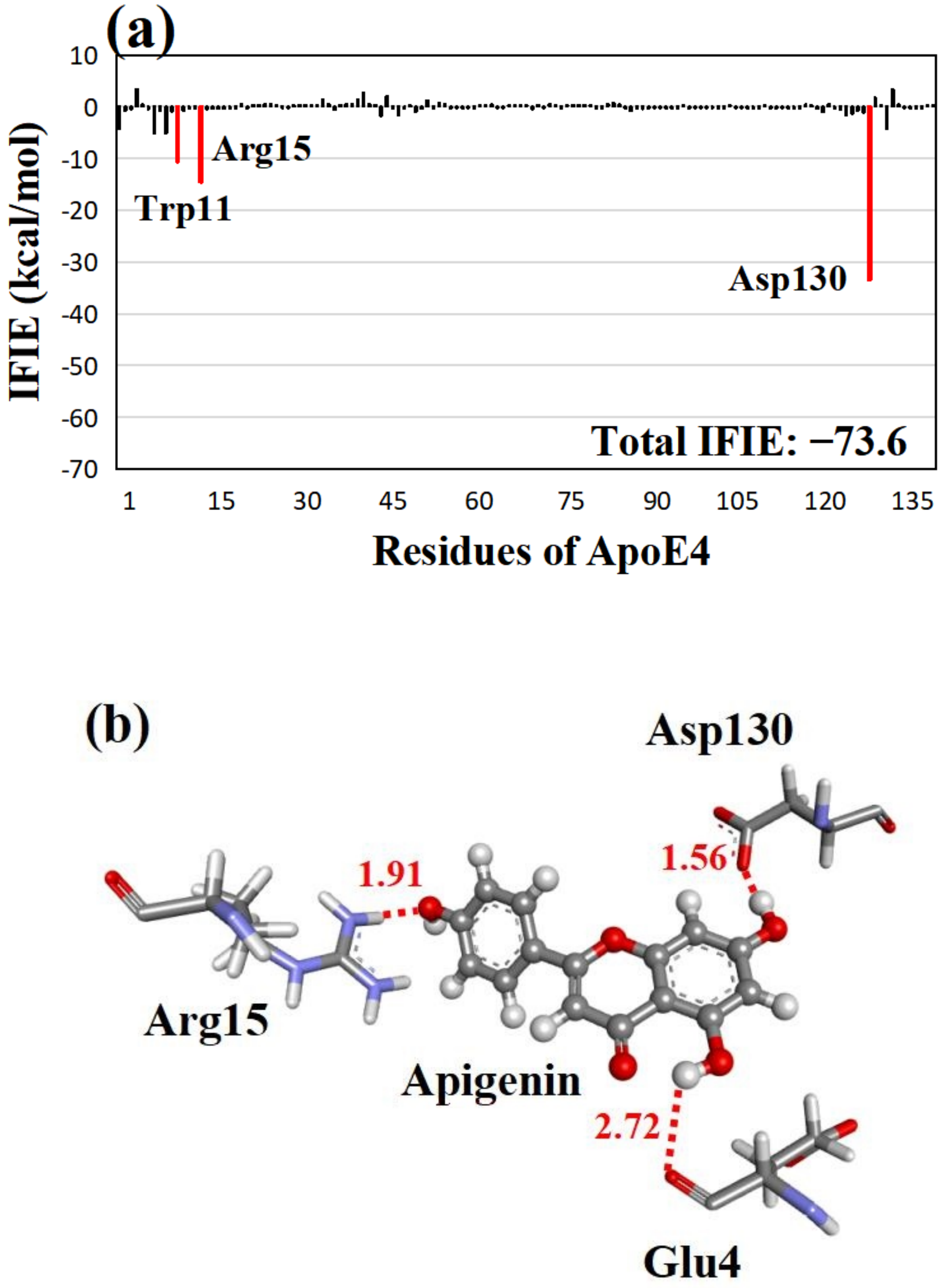

Figure S11: (a) IFIEs between the compound kaempferol and ApoE4 residues. The total IFIE of kaempferol is also shown. ApoE4 residues with negative IFIEs greater than 10 kcal/mol are displayed with red bars. **(b)** Structure of the interactions between kaempferol and key ApoE4 residues. The hydrogen bonds are shown as red dotted lines and their distances are given in Å.

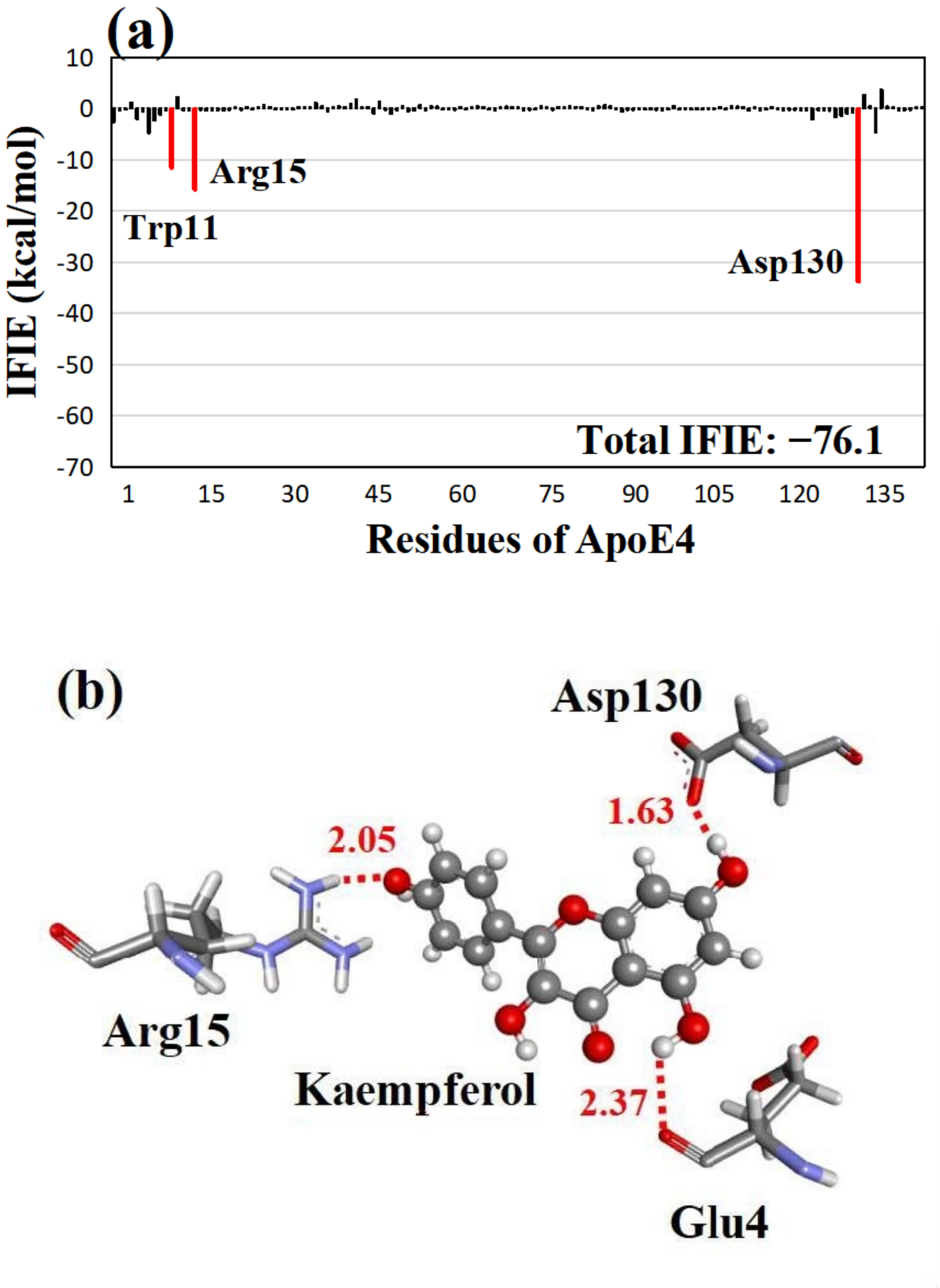

Figure S12: (a) IFIEs between the compound sinapic acid and ApoE4 residues. The total IFIE of sinapic acid is also shown. ApoE4 residues with negative IFIEs greater than 10 kcal/mol are displayed with red bars. **(b)** Structure of the interactions between sinapic acid and key ApoE4 residues. The hydrogen bonds are shown as red dotted lines and their distances are given in Å.

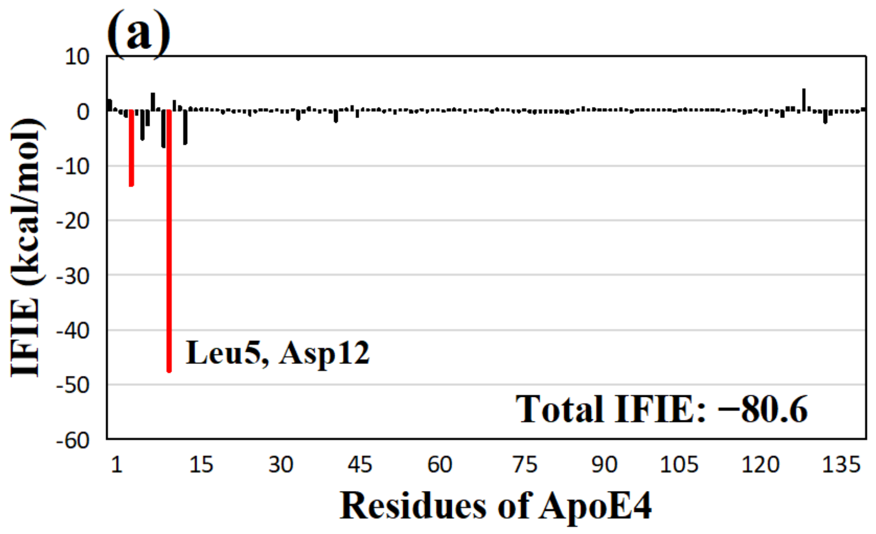

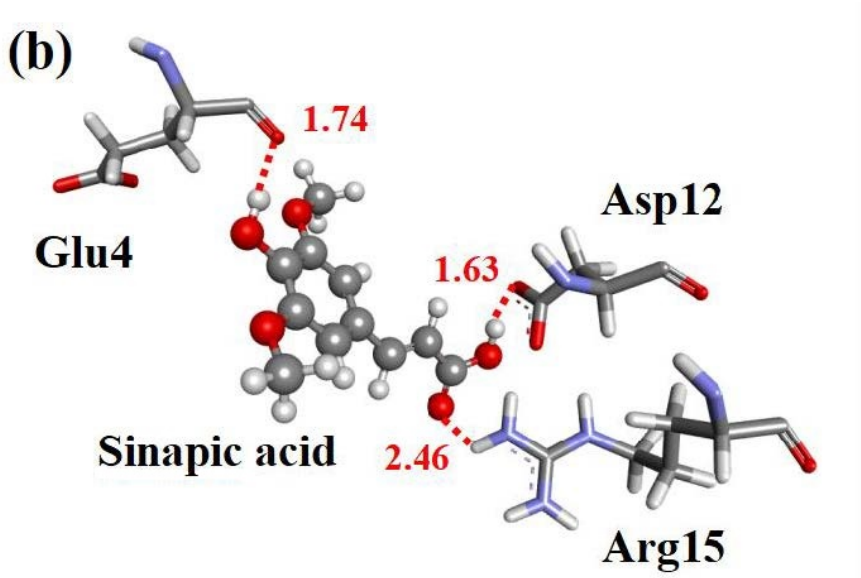

Figure S13: (a) IFIEs between the compound gallic acid and ApoE4 residues. The total IFIE of gallic acid is also shown. ApoE4 residue with negative IFIE greater than 10 kcal/mol is displayed with a red bar. **(b)** Structure of the interactions between gallic acid and key ApoE4 residues. The hydrogen bonds are shown as red dotted lines and their distances are given in Å.

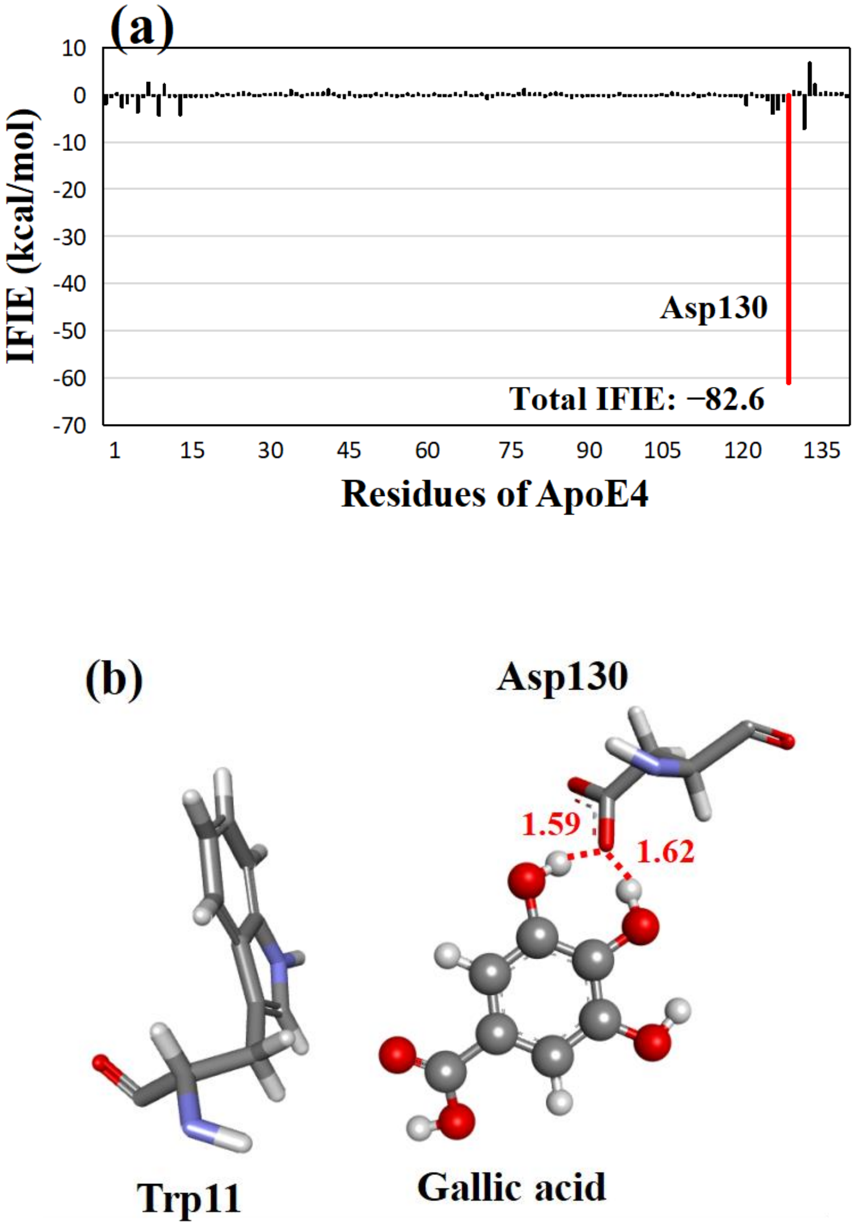

Figure S14: (a) IFIEs between the compound daidzein and ApoE4 residues. The total IFIE of daidzein is also shown. ApoE4 residues with negative IFIEs greater than 10 kcal/mol are displayed with red bars. **(b)** Structure of the interactions between daidzein and key ApoE4 residues. The hydrogen bonds are shown as red dotted lines and their distances are given in Å.

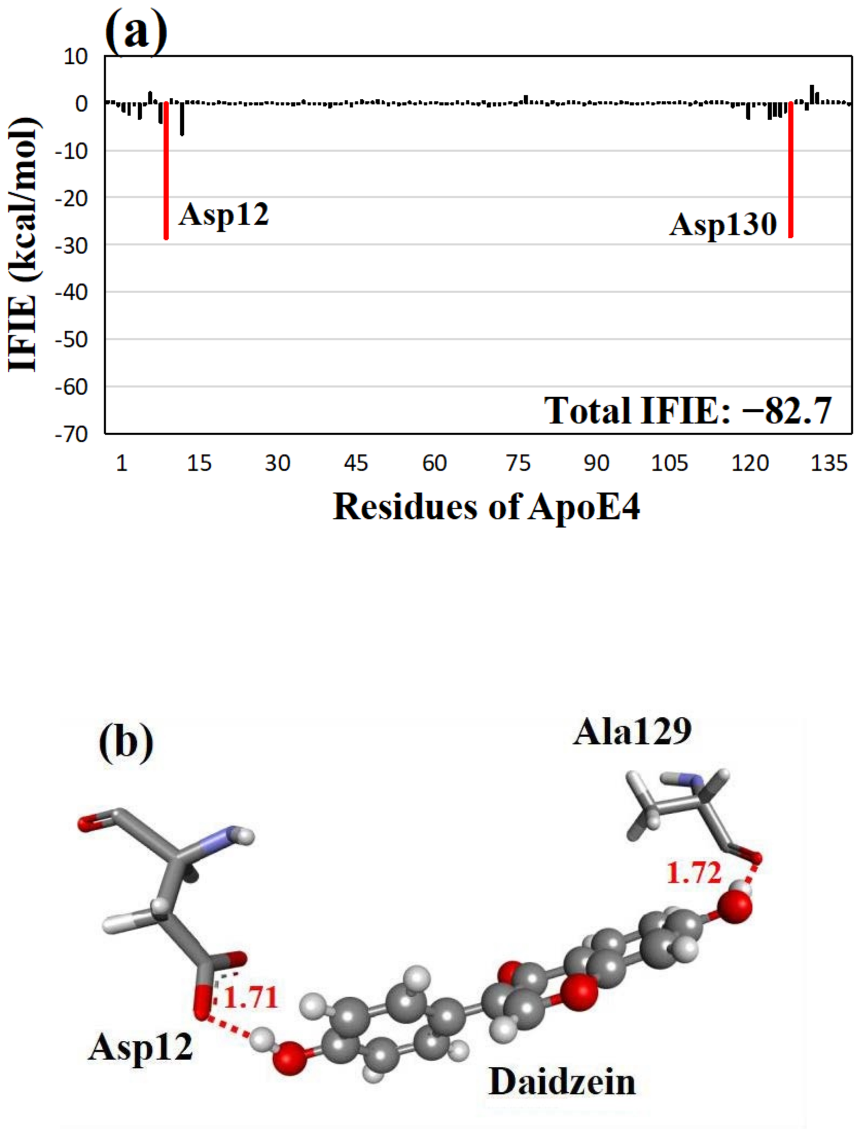

Figure S15. Classification of 26 natural compounds using visualized cluster analysis (VISCANA) based on protein-ligand interactions [69]. Each compound is indicated on the vertical axis. The horizontal axis on the right represents the ApoE4 residues existing within 10 Å of the compound. The red and blue colors indicate attractive and repulsive interactions, respectively, and the shading represents the magnitude of IFIE. The tree on the left represents the clustering of compounds based on their IFIEs with ApoE4 residues.

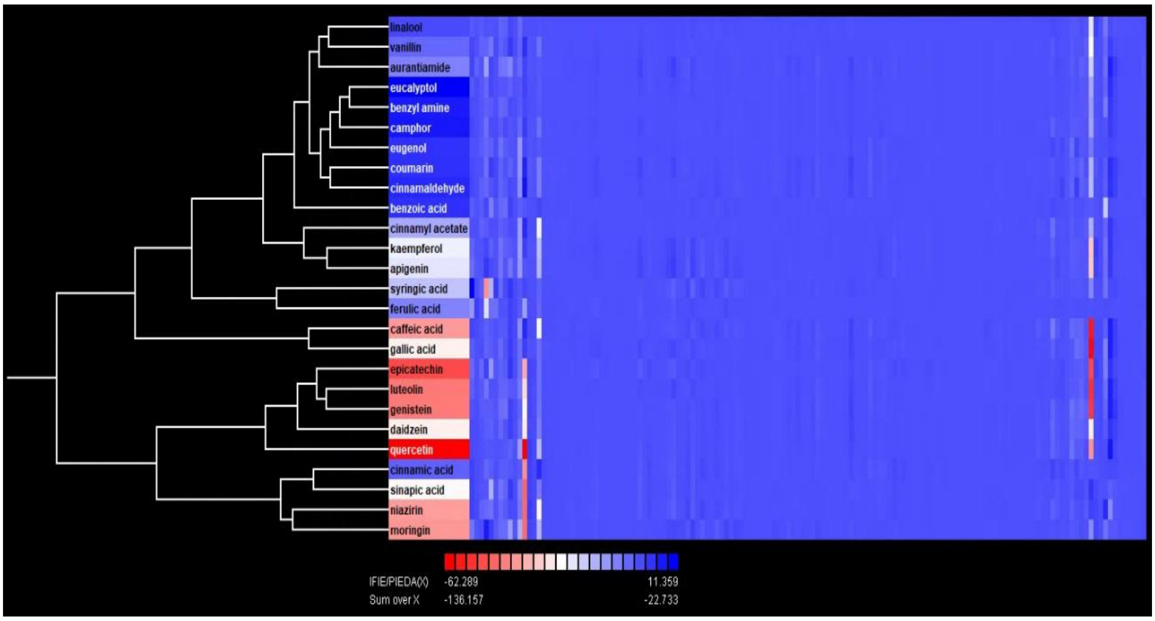

Figure S16: (a) IFIEs between the quercetin derivative **Qc** and ApoE4 residues. The total IFIE of **Qc** is also shown. ApoE4 residues with negative IFIEs greater than 10 kcal/mol are displayed with red bars. **(b)** Structure of the interactions between **Qc** and key ApoE4 residues. Hydrogen bonds are represented by red dotted lines and their distances are given in Å. The introduced hydroxyl group at the **c**-site of quercetin is shown in a blue dotted circle.

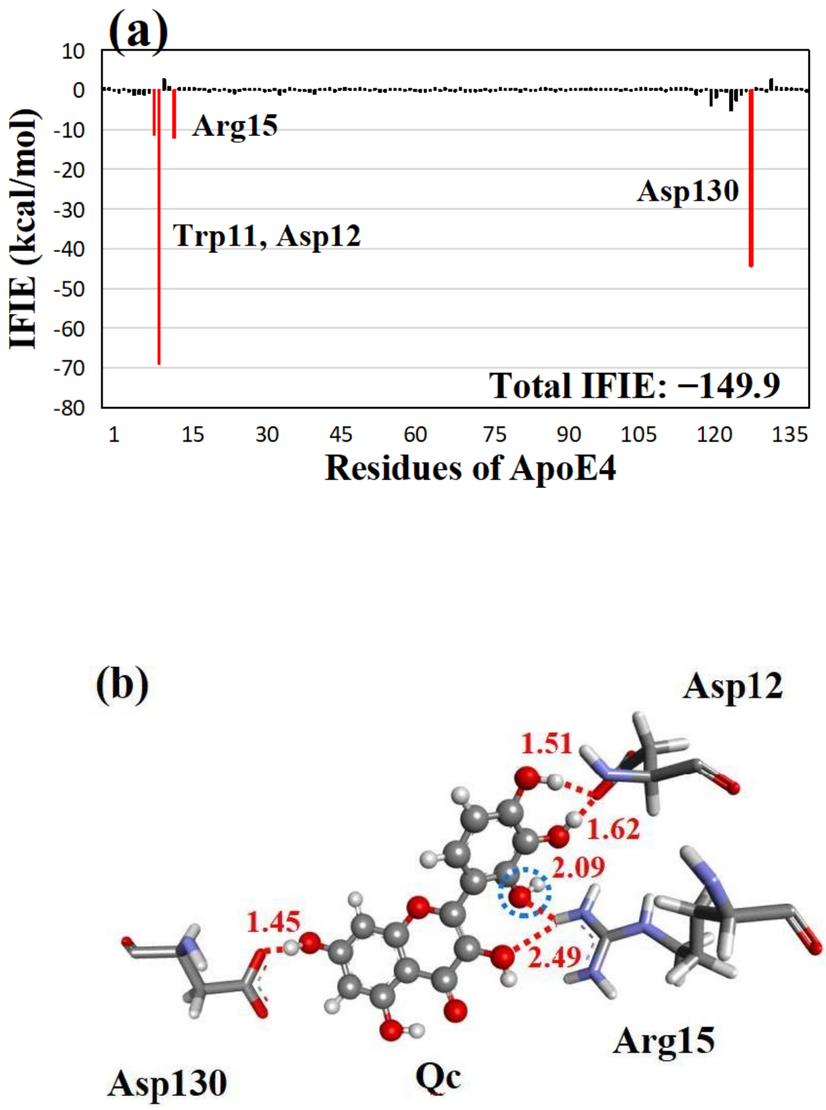

Figure S17: (a) IFIEs between the quercetin derivative **Qa** and ApoE4 residues. The total IFIE of **Qa** is also shown. ApoE4 residues with negative IFIEs greater than 10 kcal/mol are displayed with red bars. **(b)** Structure of the interactions between **Qa** and key ApoE4 residues. Hydrogen bonds are represented by red dotted lines and their distances are given in Å. The introduced hydroxyl group at the **a**-site of quercetin is shown in a blue dotted circle.

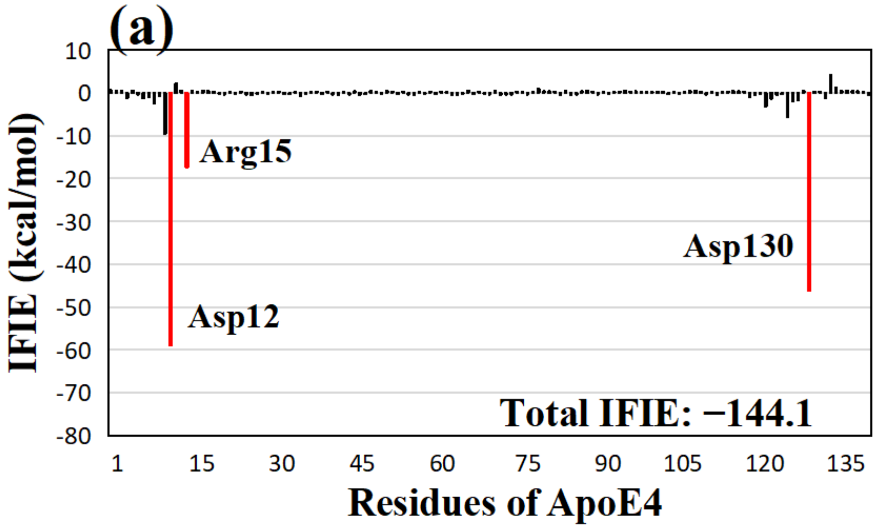

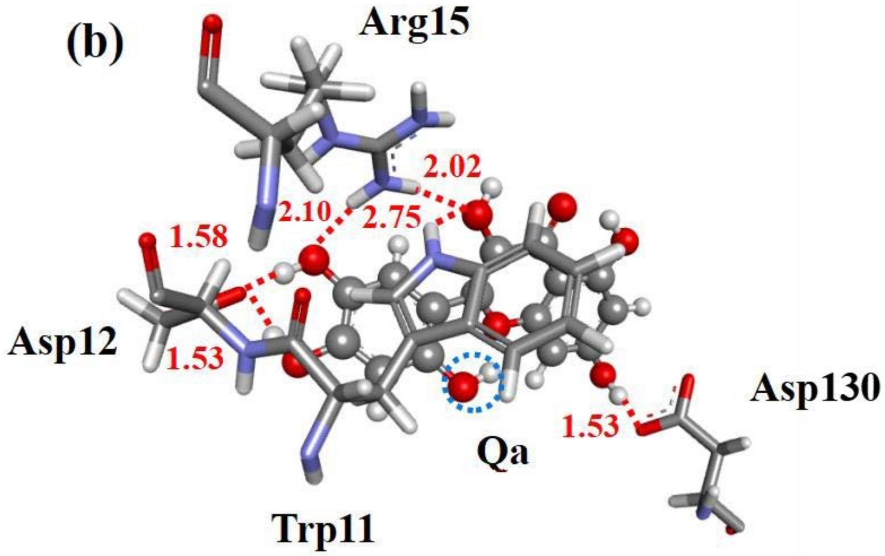

Figure S18: (a) IFIEs between the quercetin derivative **Qb** and ApoE4 residues. The total IFIE of **Qb** is also shown. ApoE4 residues with negative IFIEs greater than 10 kcal/mol are displayed with red bars. **(b)** Structure of the interactions between **Qb** and key ApoE4 residues. Hydrogen bonds are represented by red dotted lines and their distances are given in Å. The introduced hydroxyl group at the **b**-site of quercetin is shown in a blue dotted circle.

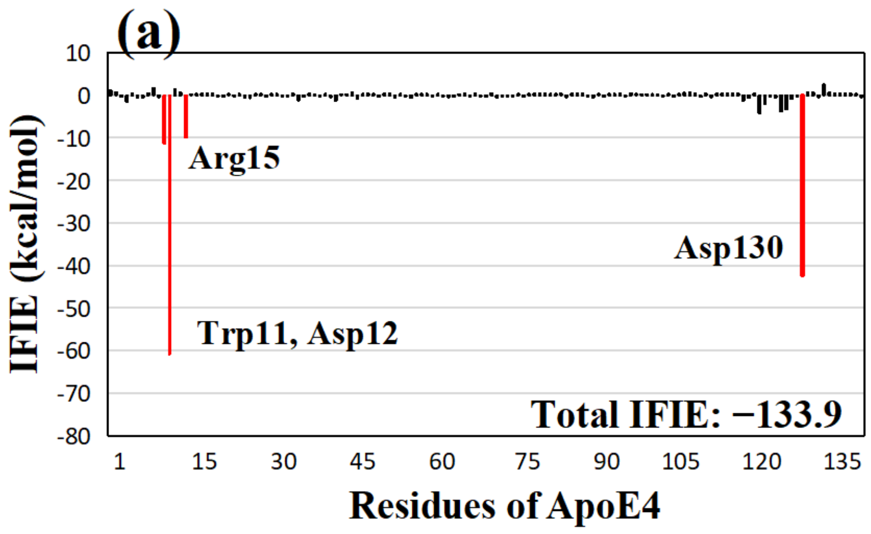

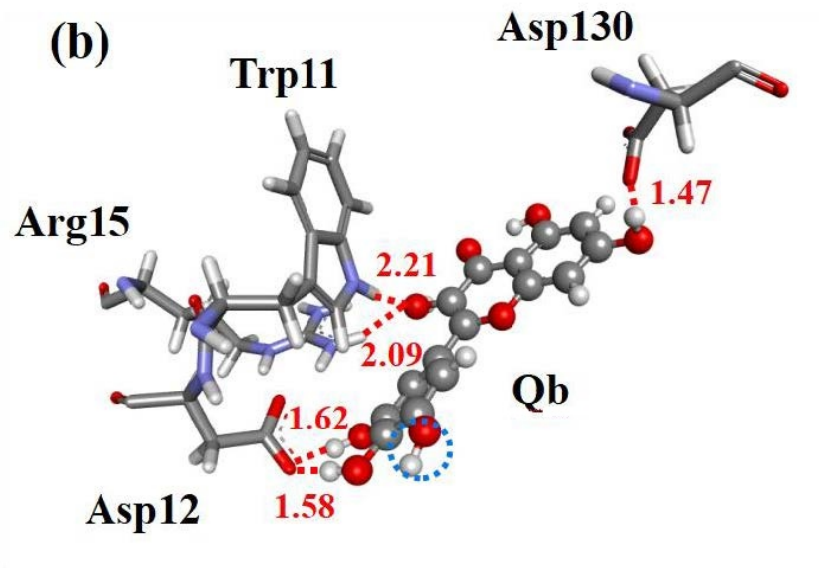

Table S1. Chemical properties of the 16 compounds contained in *Moringa oleifera* [11]. Their PubChem ID, molecular weight (MW), number of rotatable bonds (RB), number of H-bond acceptors (HBA), number of H-bond donors (HBD), octanol-water partition coefficient (LogP), and total polar surface area (TPSA) were computed using SwissADME web tool [35]. The number of rotatable bonds (RB) is counted by omitting the bonds connected to the hydrogen atoms.

Compound	PubChem ID	MW (g/mol)	RB	HBA	HBD	LogP	TPSA ($Å^2$)
(1) Benzyl amine	7504	107.15	1	1	1	1.31	26.02
(2) Vanillin	1183	152.15	2	3	1	1.20	46.53
(3) Eugenol	3314	164.20	3	2	1	2.25	29.46
(4) Gallic acid	370	170.1	1	5	4	0.21	97.99
(5) Syringic acid	10742	198.17	3	5	2	0.99	75.99
(6) Ferulic acid	445858	194.18	3	4	2	1.36	66.76
(7) Sinapic acid	637775	224.21	4	5	2	1.31	75.99
(8) Niazirin	129556	279.3	3	6	3	0.20	102.94
(9) Moringin	14865502	311.35	4	6	3	1.31	123.60
(10) Daidzein	5281708	254.24	1	4	2	2.24	70.67
(11) Genistein	5280961	270.24	1	5	3	2.04	90.90
(12) Apigenin	5280443	270.24	1	5	3	2.11	90.90
(13) Kaempferol	5280863	286.2	1	6	4	1.58	111.13
(14) Luteolin	5280445	286.24	1	6	4	1.73	111.13
(15) Quercetin	5280343	302.2	1	7	5	1.23	131.36
(16) Aurantiamide acetate	124319	444.52	13	4	2	3.89	84.50
